# Supplementary material for: Early Aspirin Discontinuation Following Acute Coronary Syndrome or Percutaneous Coronary Intervention: A Systematic Review and Meta-Analysis of Randomized Controlled Trials
Source: J Clin Med. 2020 Mar 3;9(3):680. doi: 10.3390/jcm9030680 (PMC7141190; doi:10.3390/jcm9030680)
Supplement: Supplementary file 1 [file jcm-09-00680-s001.pdf]

Supplementary file

Supplementary Figures titles and legends

Supplementary Figure S1. Flow diagram of the study selection process
M-H: Mantel Haenszel; CI: confidence interval; DAPT: dual antiplatelet therapy

Supplementary Figure S2. Estimated risk of definite or probable stent thrombosis
Same abbreviations as Online Figure 1

Supplementary Figure S3. Estimated risk of ischemic stroke
Same abbreviations as Online Figure 1

Supplementary Figure S4. Estimated risk of safety event according to fixed effect model
Same abbreviations as Online Figure 1

Supplementary Figure S5. Estimated risk of efficacy event according to fixed effect model
Same abbreviations as Online Figure 1

Supplementary Figure S6. Estimated risk of safety events after exclusion of RCTs without homogenous background OAC between the compared groups
M-H: Mantel Haenszel; CI: confidence interval; DAPT: dual antiplatelet therapy; OAC: oral anticoagulation; RCT: randomized controlled trial

Supplementary Figure S7. Estimated risk of safety event according to the BARC classification
M-H: Mantel Haenszel; CI: confidence interval; DAPT: dual antiplatelet therapy; BARC: Bleeding Academic Research Consortium

Supplementary Figure S8. Estimated risk of safety event according to the GUSTO classification
M-H: Mantel Haenszel; CI: confidence interval; DAPT: dual antiplatelet therapy: GUSTO: Global Use of Strategies to Open Occluded Arteries

Supplementary Figure S9. Estimated risk of safety event according to the ISTH classification
M-H: Mantel Haenszel; CI: confidence interval; DAPT: dual antiplatelet therapy: ISTH: International Society on Thrombosis and Haemostasis

Supplementary Figure S10. Estimated risk of safety event according to the TIMI classification
M-H: Mantel Haenszel; CI: confidence interval; DAPT: dual antiplatelet therapy; TIMI: Thrombolysis in Myocardial Infarction

Supplementary Figure S11. Estimated risk of safety event (A) and efficacy events (B) according to the $P2Y_{12}$ inhibitors predominantly used in trials without indication for chronic oral anticoagulation
Same abbreviations as Online Figure 1; MACCE: major adverse cardiac and cerebrovascular events

Supplementary Figure S12. Estimated risk of safety event(A) and efficacy events (B) according to the DAPT duration prior to aspirin discontinuation in trials without indication for chronic oral anticoagulation
Same abbreviations as Online Figure 1

Supplementary Figure S13. Estimated risk of Myocardial infarction using site-reported events with the GLOBAL LEADERS trial.
Same abbreviations as Online Figure 1

Supplementary Figure S14. Funnel plots
MACCE: major adverse cardiac and cerebrovascular event

Supplementary Table S1. PRISMA checklist items

Section/topic	#	Checklist item	Reported on page #
**TITLE**			
Title	1	Identify the report as a systematic review, meta-analysis, or both.	1
**ABSTRACT**			
Structured summary	2	Provide a structured summary including, as applicable: background; objectives; data sources; study eligibility criteria, participants, and interventions; study appraisal and synthesis methods; results; limitations; conclusions and implications of key findings; systematic review registration number.	1
**INTRODUCTION**			
Rationale	3	Describe the rationale for the review in the context of what is already known.	2
Objectives	4	Provide an explicit statement of questions being addressed with reference to participants, interventions, comparisons, outcomes, and study design (PICOS).	2
**METHODS**			
Protocol and registration	5	Indicate if a review protocol exists, if and where it can be accessed (e.g., Web address), and, if available, provide registration information including registration number.	2
Eligibility criteria	6	Specify study characteristics (e.g., PICOS, length of follow-up) and report characteristics (e.g., years considered, language, publication status) used as criteria for eligibility, giving rationale.	2
Information sources	7	Describe all information sources (e.g., databases with dates of coverage, contact with study authors to identify additional studies) in the search and date last searched.	2
Search	8	Present full electronic search strategy for at least one database, including any limits used, such that it could be repeated.	2
Study selection	9	State the process for selecting studies (i.e., screening, eligibility, included in systematic review, and, if applicable, included in the meta-analysis).	2
Data collection process	10	Describe method of data extraction from reports (e.g., piloted forms, independently, in duplicate) and any processes for obtaining and confirming data from investigators.	2

Data items	11	List and define all variables for which data were sought (e.g., PICOS, funding sources) and any assumptions and simplifications made.	2 and table 1 and 2
Risk of bias in individual studies	12	Describe methods used for assessing risk of bias of individual studies (including specification of whether this was done at the study or outcome level), and how this information is to be used in any data synthesis.	2
Summary measures	13	State the principal summary measures (e.g., risk ratio, difference in means).	3
Synthesis of results	14	Describe the methods of handling data and combining results of studies, if done, including measures of consistency (e.g., I^2) for each meta-analysis.	3

Section/topic	#	Checklist item	Reported on page #
Risk of bias across studies	15	Specify any assessment of risk of bias that may affect the cumulative evidence (e.g., publication bias, selective reporting within studies).	3
Additional analyses	16	Describe methods of additional analyses (e.g., sensitivity or subgroup analyses, meta-regression), if done, indicating which were pre-specified.	3
**RESULTS**			
Study selection	17	Give numbers of studies screened, assessed for eligibility, and included in the review, with reasons for exclusions at each stage, ideally with a flow diagram.	Online figure 1
Study characteristics	18	For each study, present characteristics for which data were extracted (e.g., study size, PICOS, follow-up period) and provide the citations.	Table 2
Risk of bias within studies	19	Present data on risk of bias of each study and, if available, any outcome level assessment (see item 12).	Online Table 4
Results of individual studies	20	For all outcomes considered (benefits or harms), present, for each study: (a) simple summary data for each intervention group (b) effect estimates and confidence intervals, ideally with a forest plot.	10-11 and figures 1-3
Synthesis of results	21	Present results of each meta-analysis done, including confidence intervals and measures of consistency.	10-11 and figures 1-3

Risk of bias across studies	22	Present results of any assessment of risk of bias across studies (see Item 15).	Online Table 4 and Online Figure 15
Additional analysis	23	Give results of additional analyses, if done (e.g., sensitivity or subgroup analyses, meta-regression [see Item 16]).	11 and Online Figure 4 to 14
**DISCUSSION**			
Summary of evidence	24	Summarize the main findings including the strength of evidence for each main outcome; consider their relevance to key groups (e.g., healthcare providers, users, and policy makers).	12
Limitations	25	Discuss limitations at study and outcome level (e.g., risk of bias), and at review-level (e.g., incomplete retrieval of identified research, reporting bias).	13
Conclusions	26	Provide a general interpretation of the results in the context of other evidence, and implications for future research.	13
**FUNDING**			
Funding	27	Describe sources of funding for the systematic review and other support (e.g., supply of data); role of funders for the systematic review.	14

Supplementary Table S2. Definitions of major, non-major and all bleeding used in each trial

Trials	Definition used for Major bleeding	Definition used for non-major bleeding	Definition used for all bleeding
**GLOBAL LEADERS**[1]	BARC 3 or 5	BARC 2	BARC 2 to 5
**STOP DAPT-2**[2]	TIMI major bleeding	TIMI minor bleeding	TIMI major or minor bleeding
**SMART CHOICE**[3]	BARC 3 or 5	BARC 2, not detailed	BARC 2 to 5
**WOEST**[4]	TIMI major bleeding	BARC 2	TIMI major or minor bleeding
**AUGUSTUS**[5]	ISTH major bleeding	CRNM ISTH bleeding	Major or CRNM ISTH bleeding
**TWILIGHT**[6]	BARC 3 or 5	BARC2, not detailed	BARC 2, 3 or 5
**REDUAL-PCI**[7]	ISTH major bleeding	CRNM ISTH bleeding, not detailed	Major or CRNM ISTH bleeding
**PIONEER-AF**[8]	TIMI major bleeding	TIMI minor bleeding	Clinically significant bleeding (major or minor TIMI bleeding or bleeding requiring medical attention)
**ENTRUST-AF**[9]	ISTH major bleeding	CRNM ISTH bleeding	Major or CRNM ISTH bleeding

BARC: Bleeding Academic Research Consortium; TIMI: Thrombolysis in Myocardial Infarction; ISTH: International Society on Thrombosis and Haemostasis; CRNM: clinically relevant non-major bleeding

Supplementary Table S3. Definitions of major adverse cardiac and cerebrovascular events in each trial

Trials	Major adverse cardiac and cerebrovascular event
**GLOBAL LEADERS**[1]	All-cause death, stroke or new Q wave myocardial infarction
**STOP DAPT-2**[2]	Cardiovascular death, myocardial infarction, definite stent thrombosis or any stroke
**SMART CHOICE**[3]	All-cause death, myocardial infarction or stroke
**WOEST**[4]	All-cause death, myocardial infarction, target vessel revascularization, stroke or stent thrombosis
**AUGUSTUS**[5]	All-cause death, myocardial infarction, stroke, definite or probable stent thrombosis or urgent revascularization
**TWILIGHT**[6]	All-cause death, myocardial infarction or stroke
**REDUAL-PCI**[7]	All-cause death, myocardial infarction, stroke, systemic embolism or unplanned revascularization
**PIONEER-AF**[8]	Cardiovascular death, myocardial infarction, stroke or stent thrombosis
**ENTRUST-AF**[9]	Cardiovascular death, myocardial infarction, stroke, systemic embolism or definite stent thrombosis

Supplementary Table S4. Procedural characteristics

Study	Radial artery access site	Number of treated lesions per patient	Lesion localization	Overall stent length (mm)
**WOEST**	145/563 (25.8%)	Not provided	LAD: 229/560 (40.9%)<br>LCX: 135/560 (24.1%)<br>RCA: 164/560 (29.3%)<br>Bypass graft: 32/560 (5.7%)	EAD: 23.4 ± 13.0<br>DAPT: 24.0 ± 12.7
**PIONEER AF-PCI**	not provided			
**REDUAL-PCI**	2.212/3.489 (63.4%)	not provided	not provided	not provided
**GLOBAL LEADERS**	11,761/15,968 (73.7%)	1 lesion: 11,805/15,818 (74.6%)<br>2 lesions: 3.187/15,818 (20.1%)<br>≥3 lesions: 826/15,818 (5.3%)	LM: 387/20,841 (1.9%)<br>LAD: 8,666/20,841 (41.6%)<br>LCX: 5,077/20,841 (24.4%)<br>RCA: 6,490/20,841 (31.1%)<br>Bypass graft: 221/20,841 (1%)	EAD: 24.8 ± 13.9<br>DAPT: 24.8 ± 14.0
**AUGUSTUS**	not provided			
**STOPDAPT-2**	2,496/3,009 (83.0%)	EAD: 1.1 ± 0.4<br>DAPT: 1.1 ± 0.4	LM: 80 (2.7%)<br>LAD: 1,682 (55.9%)<br>LCX: 573 (19.0%)<br>RCA: 846 (28.1%)	EAD: 30.3 ± 16.7<br>DAPT: 30.5 ± 16.8
**SMART CHOICE**	2.182/2.993 (72.9%)	1 lesion: 2,106/2,993 (70.4%)<br>2 lesions: 680/2,993 (22.7%)<br>≥3 lesions: 207/2,993 (6.9%)	LM: 58/3,734 (1.6%)<br>LAD: 1,853/3,734 (49.6%)<br>LCX: 775/3,734 (20.8%)<br>RCA: 1,048/3,734 (28.0%)	EAD: 38.0 ± 22.5<br>DAPT: 37.8 ± 22.9
**ENTRUST-AF PCI**	not provided			
**TWILIGHT**	5.186/7.119 (72.8%)	EAD: 1.5 ± 0.7<br>DAPT: 1.5 ± 0.7	LM: 735/9.006 (8.2%)<br>LAD: 6.719/9.006 (74.6%)<br>LCX: 4.216/9.006 (46.8%)<br>RCA: 4.807/9,006 (53.4%)	EAD: 40.1 ± 24.2<br>DAPT: 39.7 ± 24.3

LM: left main; LAD: left ascending descending coronary artery; LCX: left circumflex coronary artery; RCA: right coronary artery; EAD: Early aspirin discontinuation; DAPT: dual antiplatelet therapy

Supplementary Table S5. Bias assessment of the included studies

	TRIALS								
**Bias type**	WOEST [4]	PIONEER AF [8]	REDUAL-PCI [7]	GLOBAL LEADERS[1]	AUGUSTUS [5]	STOPDAPT-2[2]	SMART-CHOICE[3]	ENTRUST-AF-PCI[9]	TWILIGHT[6]
**Selection bias**									
Random sequence generation	Low risk	Low risk	Low risk	Low risk	Low risk	Low risk	Low risk	Low risk	Low risk
Allocation concealment	Open label	Open label	Open label	Open label	Low risk	Open label	Open label	Open label	Low risk
**Performance bias**									
Blinding of participants and personnel	Open label	Open label	Open label	Open label	Low risk	Open label	Open label	Open label	Low risk
**Detection bias**									
Blinding of outcome assessment	Low risk	Low risk	Low risk	Low risk	Low risk	Low risk	Low risk	Low risk	Low risk
**Attrition bias**									

Incomplete outcome data	Low risk	Low risk	Low risk	Low risk	Low risk	Low risk	Low risk	Low risk	Low risk
**Reporting bias**									
Selective reporting	Low risk	Low risk	Low risk	Intermediate risk	Low risk	Low risk	Low risk	Low risk	Low risk

Supplementary Figure S1. Flow diagram of the study selection process

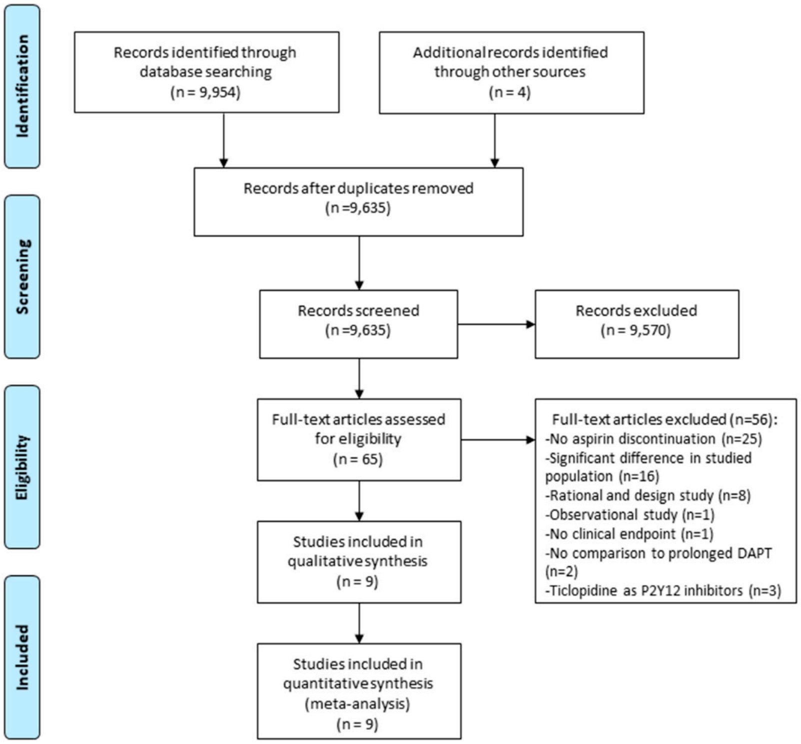

Supplementary Figure S2. Estimated risk of definite or probable stent thrombosis

Study or Subgroup	Early aspirin removal Events	Early aspirin removal Total	Dual antiplatelet therapy Events	Dual antiplatelet therapy Total	Weight	Risk Ratio M-H, Random, 95% CI
1.4.1 With chronic oral anticoagulation						
AUGUSTUS	21	2307	11	2307	38.9%	1.91 [0.92, 3.95]
Subtotal (95% CI)		2307		2307	38.9%	1.91 [0.92, 3.95]
Total events	21		11			
Heterogeneity: Not applicable						
Test for overall effect: Z = 1.74 (P = 0.08)						
1.4.2 Without chronic oral anticoagulation						
SMART CHOICE	3	1495	2	1498	11.8%	1.50 [0.25, 8.98]
STOP DAPT 2	4	1500	1	1509	8.4%	4.02 [0.45, 35.96]
TWILIGHT	14	3524	19	3515	40.9%	0.73 [0.37, 1.46]
Subtotal (95% CI)		6519		6522	61.1%	1.04 [0.46, 2.38]
Total events	21		22			
Heterogeneity: Tau² = 0.13; Chi² = 2.46, df = 2 (P = 0.29); I² = 19%						
Test for overall effect: Z = 0.10 (P = 0.92)						
Total (95% CI)		8826		8829	100.0%	1.34 [0.68, 2.62]
Total events	42		33			
Heterogeneity: Tau² = 0.17; Chi² = 4.73, df = 3 (P = 0.19); I² = 37%						
Test for overall effect: Z = 0.85 (P = 0.40)						
Test for subgroup differences: Chi² = 1.16, df = 1 (P = 0.28), I² = 13.8%						

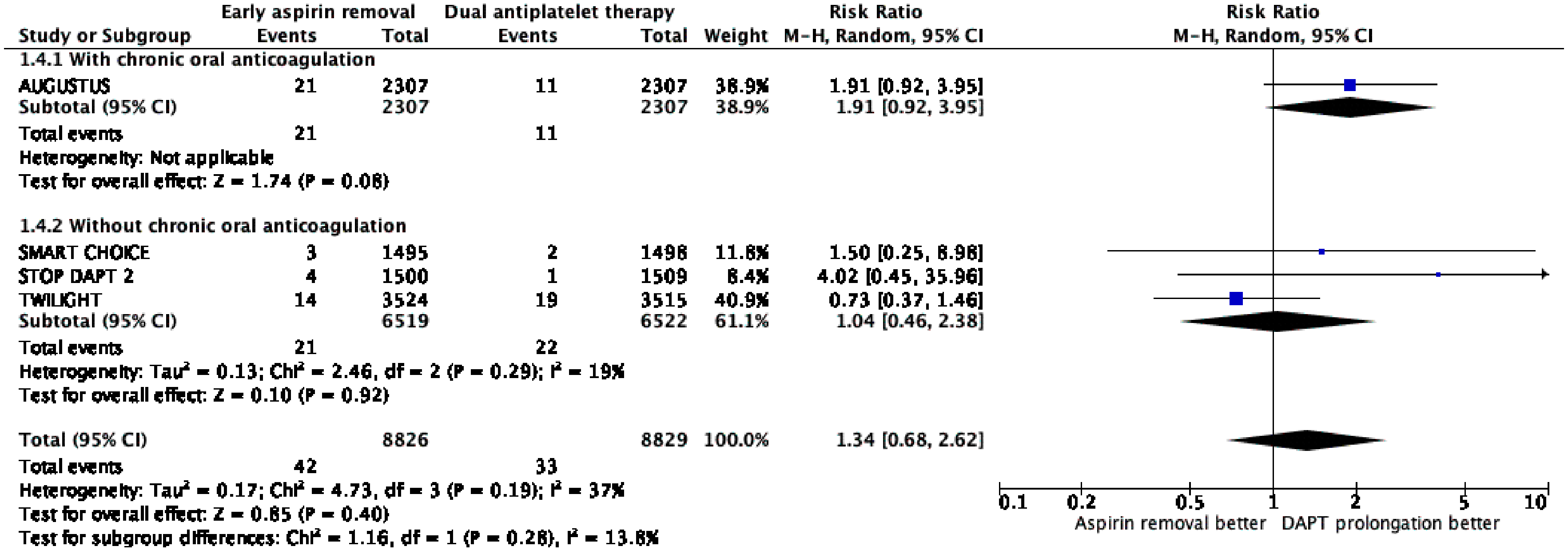

Supplementary Figure S3. Estimated risk of ischemic stroke

	Early aspirin removal		Dual antiplatelet therapy			Risk Ratio
**Study or Subgroup**	**Events**	**Total**	**Events**	**Total**	**Weight**	**M-H, Random, 95% CI**
**1.25.1 With oral anticoagulation**						
ENTRUST-AF-PCI	6	751	6	755	11.8%	1.01 [0.33, 3.10]
PIONEER AF	7	694	8	1399	13.7%	1.76 [0.64, 4.84]
WOEST	2	279	8	284	7.3%	0.25 [0.05, 1.19]
**Subtotal (95% CI)**		**1724**		**2438**	**32.7%**	**0.88 [0.32, 2.44]**
Total events	15		22			
Heterogeneity: $Tau^2 = 0.43$; $Chi^2 = 4.31$, df = 2 (P = 0.12); $I^2 = 54\%$						
Test for overall effect: Z = 0.25 (P = 0.80)						
**1.25.2 Without oral anticoagulation**						
GLOBAL LEADERS	63	7980	68	7988	33.3%	0.93 [0.66, 1.30]
STOP DAPT 2	8	1500	15	1509	16.9%	0.54 [0.23, 1.26]
TWILIGHT	16	3524	8	3515	17.1%	1.99 [0.85, 4.66]
**Subtotal (95% CI)**		**13004**		**13012**	**67.3%**	**0.98 [0.55, 1.76]**
Total events	87		91			
Heterogeneity: $Tau^2 = 0.15$; $Chi^2 = 4.68$, df = 2 (P = 0.10); $I^2 = 57\%$						
Test for overall effect: Z = 0.06 (P = 0.95)						
**Total (95% CI)**		**14728**		**15450**	**100.0%**	**0.97 [0.61, 1.53]**
Total events	102		113			
Heterogeneity: $Tau^2 = 0.13$; $Chi^2 = 8.94$, df = 5 (P = 0.11); $I^2 = 44\%$						
Test for overall effect: Z = 0.14 (P = 0.89)						
Test for subgroup differences: $Chi^2 = 0.04$, df = 1 (P = 0.85), $I^2 = 0\%$						

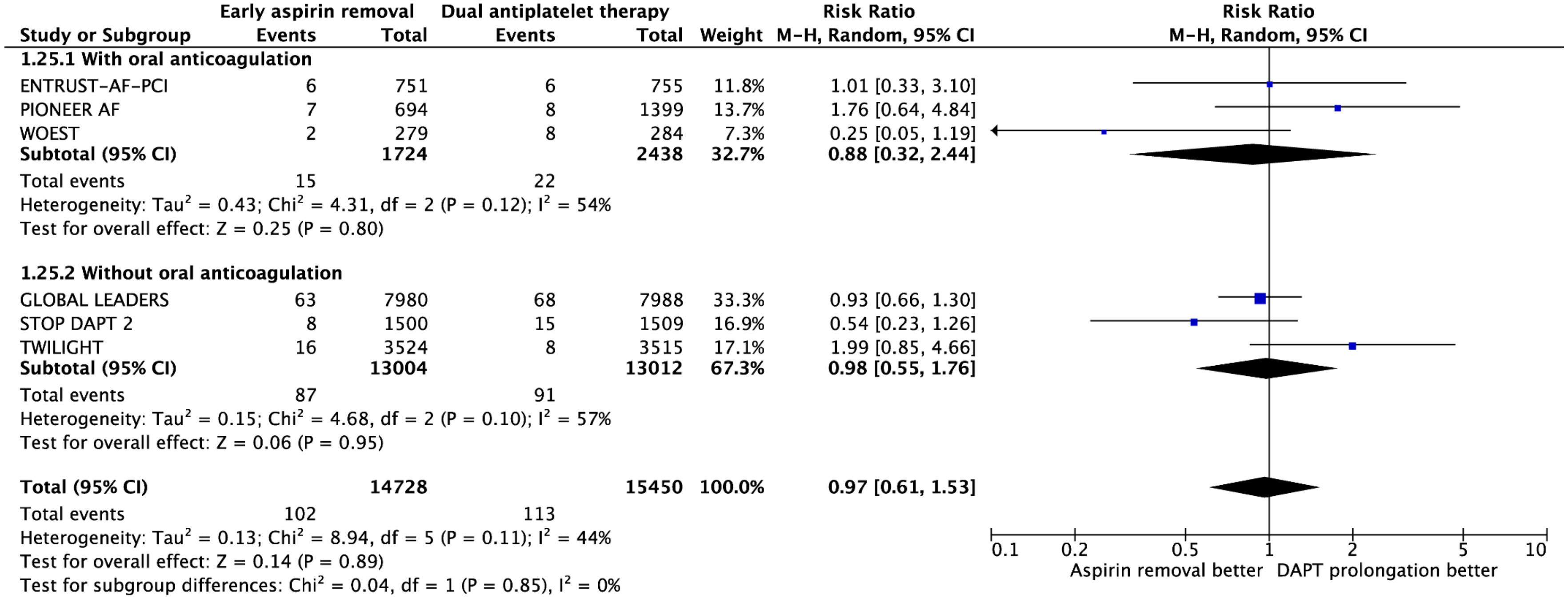

Supplementary Figure S4. Estimated risk of safety events according to fixed effect model

Major bleeding

Study or Subgroup	Early aspirin removal Events	Early aspirin removal Total	Dual antiplatelet therapy Events	Dual antiplatelet therapy Total	Weight	Risk Ratio M–H, Fixed, 95% CI
**1.7.1 With chronic oral anticoagulation**						
AUGUSTUS	65	2279	108	2277	18.8%	0.60 [0.44, 0.81]
ENTRUST–AF–PCI	45	751	48	755	8.3%	0.94 [0.64, 1.40]
PIONEER AF	14	696	32	1403	3.7%	0.88 [0.47, 1.64]
REDUAL PCI	92	1744	90	981	20.0%	0.57 [0.43, 0.76]
WOEST	9	279	16	284	2.8%	0.57 [0.26, 1.27]
**Subtotal (95% CI)**		**5749**		**5700**	**53.5%**	**0.66 [0.56, 0.79]**
Total events	225		294			
Heterogeneity: Chi² = 5.40, df = 4 (P = 0.25); I² = 26%						
Test for overall effect: Z = 4.75 (P < 0.00001)						
**1.7.2 Without chronic oral anticoagulation**						
GLOBAL LEADERS	163	7980	169	7988	29.3%	0.97 [0.78, 1.19]
SMART CHOICE	12	1495	14	1498	2.4%	0.86 [0.40, 1.85]
STOP DAPT 2	3	1500	16	1509	2.8%	0.19 [0.06, 0.65]
TWILIGHT	34	3555	69	3564	12.0%	0.49 [0.33, 0.74]
**Subtotal (95% CI)**		**14530**		**14559**	**46.5%**	**0.79 [0.66, 0.95]**
Total events	212		268			
Heterogeneity: Chi² = 13.72, df = 3 (P = 0.003); I² = 78%						
Test for overall effect: Z = 2.56 (P = 0.01)						
**Total (95% CI)**		**20279**		**20259**	**100.0%**	**0.72 [0.64, 0.82]**
Total events	437		562			
Heterogeneity: Chi² = 21.67, df = 8 (P = 0.006); I² = 63%						
Test for overall effect: Z = 5.17 (P < 0.00001)						
Test for subgroup differences: Chi² = 2.03, df = 1 (P = 0.15), I² = 50.7%						

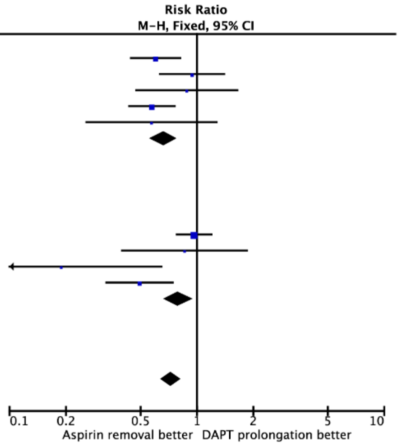

Non-major bleeding

Study or Subgroup	Early aspirin removal Events	Early aspirin removal Total	Dual antiplatelet therapy Events	Dual antiplatelet therapy Total	Weight	Risk Ratio M–H, Fixed, 95% CI
**1.8.1 With chronic oral anticoagulation**						
AUGUSTUS	148	2279	275	2277	32.0%	0.54 [0.44, 0.65]
ENTRUST–AF–PCI	97	751	114	755	13.2%	0.86 [0.67, 1.10]
PIONEER AF	7	696	20	1403	1.5%	0.71 [0.30, 1.66]
WOEST	23	279	59	284	6.8%	0.40 [0.25, 0.62]
**Subtotal (95% CI)**		**4005**		**4719**	**53.6%**	**0.60 [0.52, 0.69]**
Total events	275		468			
Heterogeneity: Chi² = 12.24, df = 3 (P = 0.007); I² = 75%						
Test for overall effect: Z = 7.01 (P < 0.00001)						
**1.8.2 WIthout chronic oral anticoagulation**						
GLOBAL LEADERS	393	7980	392	7988	45.6%	1.00 [0.88, 1.15]
STOP DAPT 2	3	1500	7	1509	0.8%	0.43 [0.11, 1.66]
**Subtotal (95% CI)**		**9480**		**9497**	**46.4%**	**0.99 [0.87, 1.14]**
Total events	396		399			
Heterogeneity: Chi² = 1.49, df = 1 (P = 0.22); I² = 33%						
Test for overall effect: Z = 0.09 (P = 0.93)						
**Total (95% CI)**		**13485**		**14216**	**100.0%**	**0.78 [0.71, 0.86]**
Total events	671		867			
Heterogeneity: Chi² = 37.49, df = 5 (P < 0.00001); I² = 87%						
Test for overall effect: Z = 4.91 (P < 0.00001)						
Test for subgroup differences: Chi² = 24.96, df = 1 (P < 0.00001), I² = 96.0%						

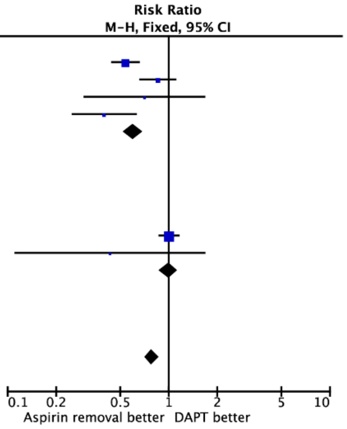

All bleeding

Study or Subgroup	Early aspirin removal Events	Early aspirin removal Total	Dual antiplatelet therapy Events	Dual antiplatelet therapy Total	Weight	Risk Ratio M–H, Fixed, 95% CI
**1.9.1 With chronic oral anticoagulation**						
AUGUSTUS	204	2279	367	2277	18.4%	0.56 [0.47, 0.65]
ENTRUST–AF–PCI	128	751	152	755	7.6%	0.85 [0.68, 1.05]
PIONEER AF	109	696	284	1403	9.5%	0.77 [0.63, 0.95]
REDUAL PCI	305	1744	264	981	17.0%	0.65 [0.56, 0.75]
WOEST	39	279	89	284	4.4%	0.45 [0.32, 0.63]
**Subtotal (95% CI)**		**5749**		**5700**	**56.9%**	**0.65 [0.60, 0.71]**
Total events	785		1156			
Heterogeneity: Chi² = 17.25, df = 4 (P = 0.002); I² = 77%						
Test for overall effect: Z = 10.05 (P < 0.00001)						
**1.9.2 Without chronic oral anticoagulation**						
GLOBAL LEADERS	535	7980	536	7988	26.9%	1.00 [0.89, 1.12]
SMART CHOICE	28	1495	49	1498	2.5%	0.57 [0.36, 0.91]
STOP DAPT 2	6	1500	23	1509	1.2%	0.26 [0.11, 0.64]
TWILIGHT	141	3555	250	3564	12.5%	0.57 [0.46, 0.69]
**Subtotal (95% CI)**		**14530**		**14559**	**43.1%**	**0.83 [0.75, 0.91]**
Total events	710		858			
Heterogeneity: Chi² = 32.74, df = 3 (P < 0.00001); I² = 91%						
Test for overall effect: Z = 3.82 (P = 0.0001)						
**Total (95% CI)**		**20279**		**20259**	**100.0%**	**0.73 [0.68, 0.77]**
Total events	1495		2014			
Heterogeneity: Chi² = 64.43, df = 8 (P < 0.00001); I² = 88%						
Test for overall effect: Z = 9.86 (P < 0.00001)						
Test for subgroup differences: Chi² = 13.82, df = 1 (P = 0.0002), I² = 92.8%						

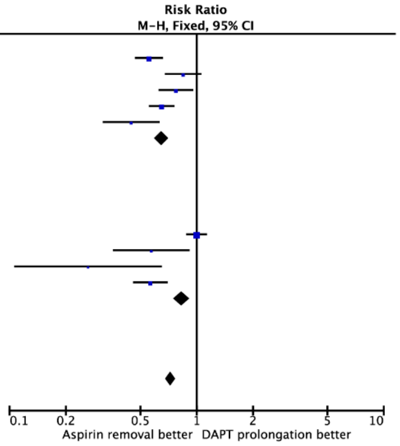

Supplementary Figure S5. Estimated risk of efficacy events according to fixed effect model

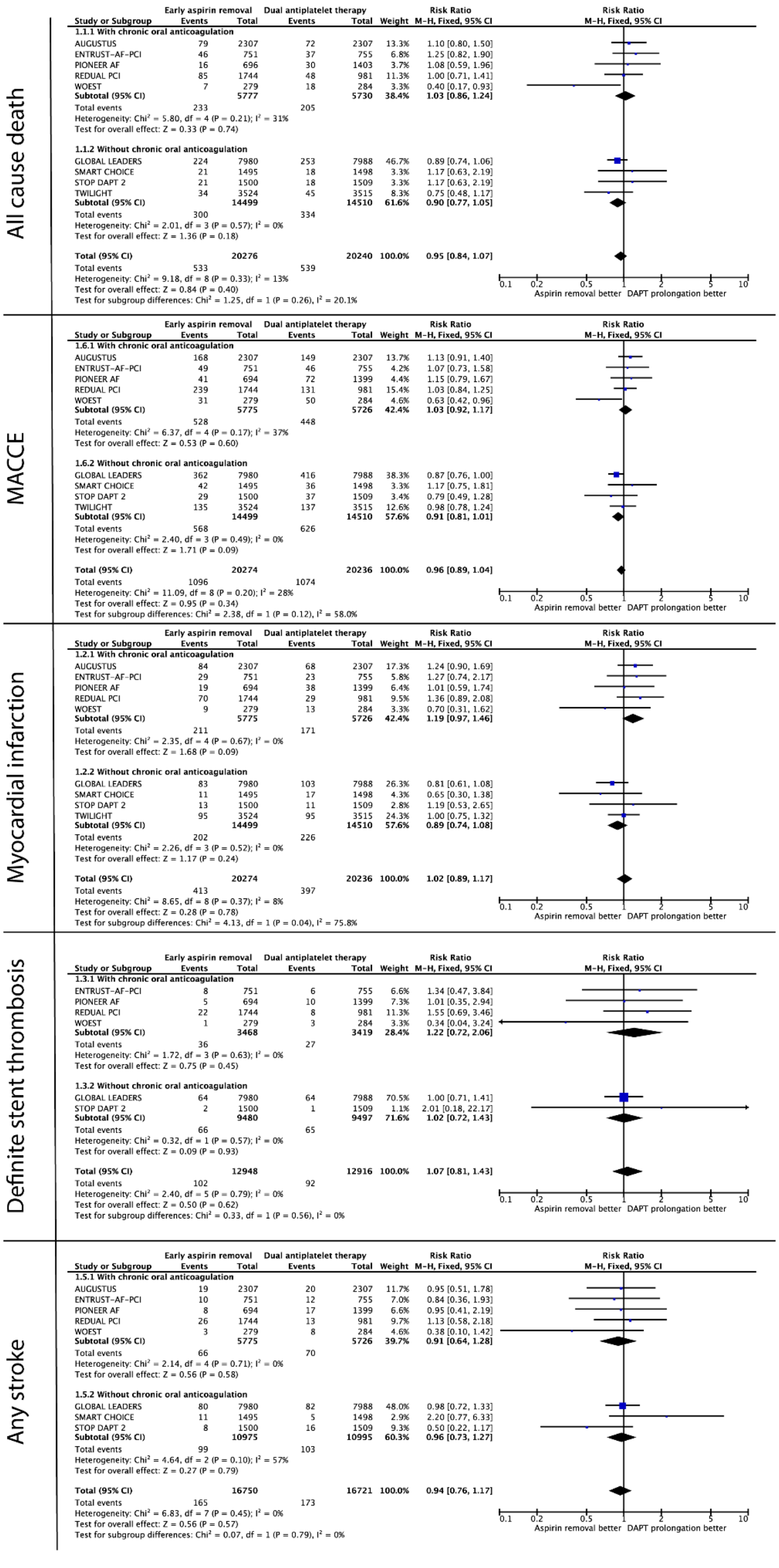

Supplementary Figure S6. Estimated risk of safety events after exclusion of RCTs without homogenous background OAC between the compared groups

Major bleeding

Study or Subgroup	Aspirin early removal Events	Aspirin early removal Total	DAPT prolongation Events	DAPT prolongation Total	Weight	Risk Ratio M-H, Random, 95% CI
**1.10.1 With chronic oral anticoagulation**						
AUGUSTUS	65	2279	108	2277	23.6%	0.60 [0.44, 0.81]
WOEST	9	279	16	284	11.5%	0.57 [0.26, 1.27]
**Subtotal (95% CI)**		**2558**		**2561**	**35.1%**	**0.60 [0.45, 0.79]**
Total events	74		124			
Heterogeneity: $Tau^2 = 0.00$; $Chi^2 = 0.01$, df = 1 (P = 0.91); $I^2 = 0\%$						
Test for overall effect: Z = 3.57 (P = 0.0004)						
**1.10.2 Without chronic oral anticoagulation**						
GLOBAL LEADERS	163	7980	169	7988	25.9%	0.97 [0.78, 1.19]
SMART CHOICE	12	1495	14	1498	12.0%	0.86 [0.40, 1.85]
STOP DAPT 2	3	1500	16	1509	6.3%	0.19 [0.06, 0.65]
TWILIGHT	34	3555	69	3564	20.6%	0.49 [0.33, 0.74]
**Subtotal (95% CI)**		**14530**		**14559**	**64.9%**	**0.62 [0.36, 1.07]**
Total events	212		268			
Heterogeneity: $Tau^2 = 0.21$; $Chi^2 = 13.72$, df = 3 (P = 0.003); $I^2 = 78\%$						
Test for overall effect: Z = 1.70 (P = 0.09)						
**Total (95% CI)**		**17088**		**17120**	**100.0%**	**0.63 [0.44, 0.89]**
Total events	286		392			
Heterogeneity: $Tau^2 = 0.11$; $Chi^2 = 16.89$, df = 5 (P = 0.005); $I^2 = 70\%$						
Test for overall effect: Z = 2.59 (P = 0.010)						
Test for subgroup differences: $Chi^2 = 0.01$, df = 1 (P = 0.90), $I^2 = 0\%$						

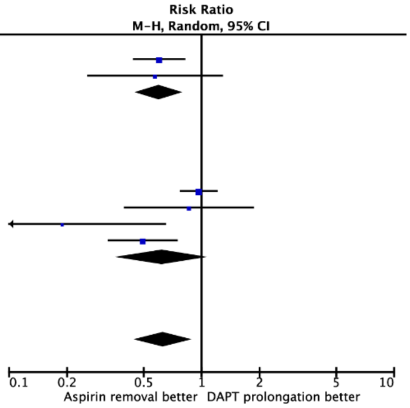

Non-major bleeding

Study or Subgroup	Early aspirin removal Events	Early aspirin removal Total	DAPT prolongation Events	DAPT prolongation Total	Weight	Risk Ratio M-H, Random, 95% CI
**1.11.1 With chronic oral anticoagulation**						
AUGUSTUS	148	2279	275	2277	31.6%	0.54 [0.44, 0.65]
WOEST	23	279	59	284	26.2%	0.40 [0.25, 0.62]
**Subtotal (95% CI)**		**2558**		**2561**	**57.8%**	**0.50 [0.38, 0.65]**
Total events	171		334			
Heterogeneity: $Tau^2 = 0.01$; $Chi^2 = 1.47$, df = 1 (P = 0.23); $I^2 = 32\%$						
Test for overall effect: Z = 5.24 (P < 0.00001)						
**1.11.2 Without chronic oral anticoagulation**						
GLOBAL LEADERS	393	7980	392	7988	32.4%	1.00 [0.88, 1.15]
STOP DAPT 2	3	1500	7	1509	9.8%	0.43 [0.11, 1.66]
**Subtotal (95% CI)**		**9480**		**9497**	**42.2%**	**0.87 [0.47, 1.62]**
Total events	396		399			
Heterogeneity: $Tau^2 = 0.12$; $Chi^2 = 1.49$, df = 1 (P = 0.22); $I^2 = 33\%$						
Test for overall effect: Z = 0.44 (P = 0.66)						
**Total (95% CI)**		**12038**		**12058**	**100.0%**	**0.59 [0.36, 0.98]**
Total events	567		733			
Heterogeneity: $Tau^2 = 0.20$; $Chi^2 = 36.96$, df = 3 (P < 0.00001); $I^2 = 92\%$						
Test for overall effect: Z = 2.02 (P = 0.04)						
Test for subgroup differences: $Chi^2 = 2.63$, df = 1 (P = 0.10), $I^2 = 62.0\%$						

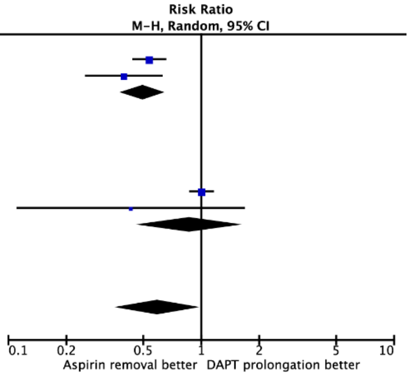

All bleeding

Study or Subgroup	Early aspirin removal Events	Early aspirin removal Total	DAPT prolongation Events	DAPT prolongation Total	Weight	Risk Ratio M-H, Random, 95% CI
**1.12.1 With chronic oral anticoagulation**						
AUGUSTUS	204	2279	367	2277	20.3%	0.56 [0.47, 0.65]
WOEST	39	279	59	284	16.6%	0.67 [0.47, 0.97]
**Subtotal (95% CI)**		**2558**		**2561**	**36.9%**	**0.57 [0.49, 0.66]**
Total events	243		426			
Heterogeneity: $Tau^2 = 0.00$; $Chi^2 = 0.87$, df = 1 (P = 0.35); $I^2 = 0\%$						
Test for overall effect: Z = 7.40 (P < 0.00001)						
**1.12.2 Without chronic oral anticoagulation**						
GLOBAL LEADERS	535	7980	536	7988	20.8%	1.00 [0.89, 1.12]
SMART CHOICE	28	1495	49	1498	14.7%	0.57 [0.36, 0.91]
STOP DAPT 2	6	1500	23	1509	7.9%	0.26 [0.11, 0.64]
TWILIGHT	141	3555	250	3564	19.7%	0.57 [0.46, 0.69]
**Subtotal (95% CI)**		**14530**		**14559**	**63.1%**	**0.61 [0.39, 0.96]**
Total events	710		858			
Heterogeneity: $Tau^2 = 0.17$; $Chi^2 = 32.74$, df = 3 (P < 0.00001); $I^2 = 91\%$						
Test for overall effect: Z = 2.15 (P = 0.03)						
**Total (95% CI)**		**17088**		**17120**	**100.0%**	**0.62 [0.45, 0.84]**
Total events	953		1284			
Heterogeneity: $Tau^2 = 0.12$; $Chi^2 = 51.51$, df = 5 (P < 0.00001); $I^2 = 90\%$						
Test for overall effect: Z = 3.01 (P = 0.003)						
Test for subgroup differences: $Chi^2 = 0.06$, df = 1 (P = 0.81), $I^2 = 0\%$						

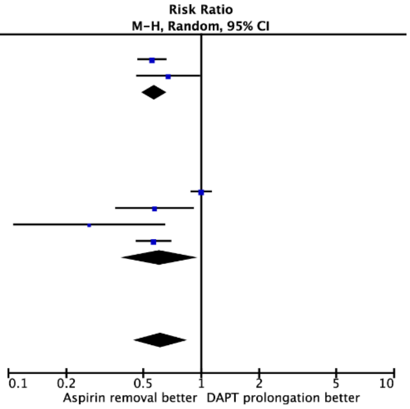

Supplementary Figure S7. Estimated risk of safety events according to the BARC classification

BARC Classification

Major bleeding

Study or Subgroup	Early aspirin removal Events	Early aspirin removal Total	DAPT prolongation Events	DAPT prolongation Total	Weight	Risk Ratio M-H, Random, 95% CI
**1.13.1 With chronic oral anticoagulation**						
ENTRUST-AF-PCI	36	751	42	755	18.5%	0.86 [0.56, 1.33]
WOEST	18	279	36	284	15.9%	0.51 [0.30, 0.87]
**Subtotal (95% CI)**		**1030**		**1039**	**34.4%**	**0.68 [0.41, 1.14]**
Total events	54		78			
Heterogeneity: Tau² = 0.08; Chi² = 2.22, df = 1 (P = 0.14); I² = 55%						
Test for overall effect: Z = 1.47 (P = 0.14)						
**1.13.2 Without chronic oral anticoagulation**						
GLOBAL LEADERS	163	7980	169	7988	23.9%	0.97 [0.78, 1.19]
SMART CHOICE	12	1495	14	1498	11.3%	0.86 [0.40, 1.85]
STOP DAPT 2	8	1500	27	1509	11.0%	0.30 [0.14, 0.65]
TWILIGHT	35	3555	69	3564	19.3%	0.51 [0.34, 0.76]
**Subtotal (95% CI)**		**14530**		**14559**	**65.6%**	**0.63 [0.38, 1.05]**
Total events	218		279			
Heterogeneity: Tau² = 0.19; Chi² = 13.96, df = 3 (P = 0.003); I² = 79%						
Test for overall effect: Z = 1.79 (P = 0.07)						
**Total (95% CI)**		**15560**		**15598**	**100.0%**	**0.65 [0.46, 0.92]**
Total events	272		357			
Heterogeneity: Tau² = 0.12; Chi² = 16.60, df = 5 (P = 0.005); I² = 70%						
Test for overall effect: Z = 2.43 (P = 0.02)						
Test for subgroup differences: Chi² = 0.05, df = 1 (P = 0.83), I² = 0%						

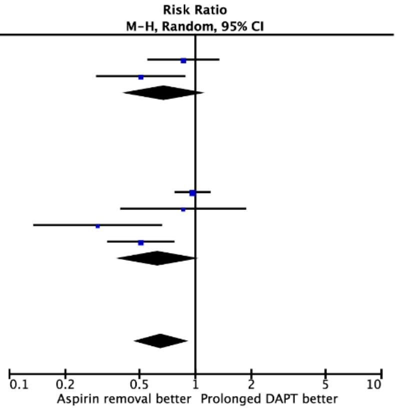

Non-major bleeding

Study or Subgroup	Early aspirin removal Events	Early aspirin removal Total	DAPT prolongation Events	DAPT prolongation Total	Weight	Risk Ratio M-H, Random, 95% CI
**1.17.1 With oral anticoagulation**						
ENTRUST-AF-PCI	101	751	112	755	25.7%	0.91 [0.71, 1.16]
PIONEER AF	92	696	217	1403	26.8%	0.85 [0.68, 1.07]
WOEST	23	279	59	284	17.0%	0.40 [0.25, 0.62]
**Subtotal (95% CI)**		**1726**		**2442**	**69.5%**	**0.71 [0.48, 1.05]**
Total events	216		388			
Heterogeneity: Tau² = 0.09; Chi² = 10.54, df = 2 (P = 0.005); I² = 81%						
Test for overall effect: Z = 1.73 (P = 0.08)						
**1.17.2 Without oral anticoagulation**						
GLOBAL LEADERS	393	7980	392	7988	30.5%	1.00 [0.88, 1.15]
**Subtotal (95% CI)**		**7980**		**7988**	**30.5%**	**1.00 [0.88, 1.15]**
Total events	393		392			
Heterogeneity: Not applicable						
Test for overall effect: Z = 0.05 (P = 0.96)						
**Total (95% CI)**		**9706**		**10430**	**100.0%**	**0.80 [0.61, 1.04]**
Total events	609		780			
Heterogeneity: Tau² = 0.06; Chi² = 15.19, df = 3 (P = 0.002); I² = 80%						
Test for overall effect: Z = 1.64 (P = 0.10)						
Test for subgroup differences: Chi² = 2.72, df = 1 (P = 0.10), I² = 63.2%						

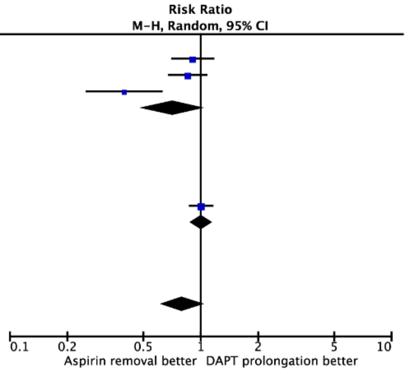

All bleeding

Study or Subgroup	Early aspirin removal Events	Early aspirin removal Total	DAPT prolongation Events	DAPT prolongation Total	Weight	Risk Ratio M-H, Random, 95% CI
**1.21.1 With oral anticoagulation**						
ENTRUST-AF-PCI	124	751	144	755	21.2%	0.87 [0.70, 1.08]
WOEST	40	279	90	284	18.6%	0.45 [0.32, 0.63]
**Subtotal (95% CI)**		**1030**		**1039**	**39.9%**	**0.63 [0.34, 1.20]**
Total events	164		234			
Heterogeneity: Tau² = 0.19; Chi² = 10.19, df = 1 (P = 0.001); I² = 90%						
Test for overall effect: Z = 1.41 (P = 0.16)						
**1.21.2 Without oral anticoagulation**						
GLOBAL LEADERS	535	7980	536	7988	22.9%	1.00 [0.89, 1.12]
SMART CHOICE	28	1495	49	1498	15.7%	0.57 [0.36, 0.91]
TWILIGHT	141	3555	250	3564	21.5%	0.57 [0.46, 0.69]
**Subtotal (95% CI)**		**13030**		**13050**	**60.1%**	**0.70 [0.45, 1.10]**
Total events	704		835			
Heterogeneity: Tau² = 0.14; Chi² = 26.15, df = 2 (P < 0.00001); I² = 92%						
Test for overall effect: Z = 1.53 (P = 0.13)						
**Total (95% CI)**		**14060**		**14089**	**100.0%**	**0.68 [0.49, 0.93]**
Total events	868		1069			
Heterogeneity: Tau² = 0.11; Chi² = 39.12, df = 4 (P < 0.00001); I² = 90%						
Test for overall effect: Z = 2.43 (P = 0.02)						
Test for subgroup differences: Chi² = 0.06, df = 1 (P = 0.80), I² = 0%						

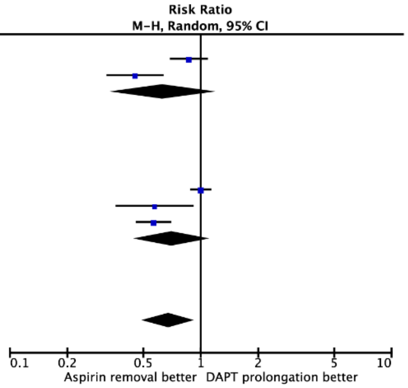

Supplementary Figure S8. Estimated risk of safety events according to the GUSTO classification

GUSTO Classification

Major bleeding

Study or Subgroup	Early aspirin removal Events	Early aspirin removal Total	DAPT prolongation Events	DAPT prolongation Total	Weight	Risk Ratio M-H, Random, 95% CI
**1.16.1 With oral anticoagulation**						
AUGUSTUS	6	2279	7	2277	21.8%	0.86 [0.29, 2.54]
PIONEER AF	7	696	30	1403	38.7%	0.47 [0.21, 1.07]
WOEST	4	279	10	284	19.6%	0.41 [0.13, 1.28]
**Subtotal (95% CI)**		**3254**		**3964**	**80.2%**	**0.53 [0.30, 0.94]**
Total events	17		47			
Heterogeneity: Tau² = 0.00; Chi² = 1.03, df = 2 (P = 0.60); I² = 0%						
Test for overall effect: Z = 2.16 (P = 0.03)						
**1.16.2 Without chronic oral anticoagulation**						
STOP DAPT 2	4	1500	11	1509	19.8%	0.37 [0.12, 1.15]
**Subtotal (95% CI)**		**1500**		**1509**	**19.8%**	**0.37 [0.12, 1.15]**
Total events	4		11			
Heterogeneity: Not applicable						
Test for overall effect: Z = 1.73 (P = 0.08)						
**Total (95% CI)**		**4754**		**5473**	**100.0%**	**0.50 [0.30, 0.82]**
Total events	21		58			
Heterogeneity: Tau² = 0.00; Chi² = 1.37, df = 3 (P = 0.71); I² = 0%						
Test for overall effect: Z = 2.70 (P = 0.007)						
Test for subgroup differences: Chi² = 0.34, df = 1 (P = 0.56), I² = 0%						

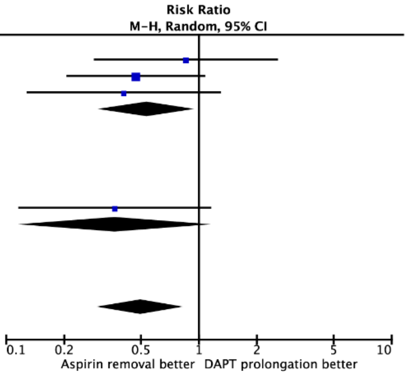

Non-major bleeding

Study or Subgroup	Early aspirin removal Events	Early aspirin removal Total	DAPT prolongation Events	DAPT prolongation Total	Weight	Risk Ratio M-H, Random, 95% CI
**1.20.1 With oral anticoagulation**						
AUGUSTUS	37	2279	63	2277	43.6%	0.59 [0.39, 0.88]
PIONEER AF	13	696	19	1403	36.6%	1.38 [0.69, 2.78]
**Subtotal (95% CI)**		**2975**		**3680**	**80.2%**	**0.86 [0.37, 1.97]**
Total events	50		82			
Heterogeneity: Tau² = 0.28; Chi² = 4.31, df = 1 (P = 0.04); I² = 77%						
Test for overall effect: Z = 0.37 (P = 0.71)						
**1.20.2 Without oral anticoagulation**						
STOP DAPT 2	2	1500	12	1509	19.8%	0.17 [0.04, 0.75]
**Subtotal (95% CI)**		**1500**		**1509**	**19.8%**	**0.17 [0.04, 0.75]**
Total events	2		12			
Heterogeneity: Not applicable						
Test for overall effect: Z = 2.34 (P = 0.02)						
**Total (95% CI)**		**4475**		**5189**	**100.0%**	**0.63 [0.26, 1.49]**
Total events	52		94			
Heterogeneity: Tau² = 0.41; Chi² = 7.87, df = 2 (P = 0.02); I² = 75%						
Test for overall effect: Z = 1.06 (P = 0.29)						
Test for subgroup differences: Chi² = 3.49, df = 1 (P = 0.06), I² = 71.3%						

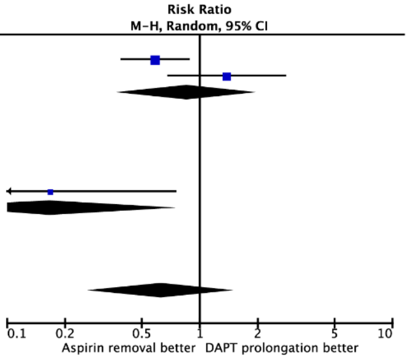

All bleeding

Study or Subgroup	Early aspirin removal Events	Early aspirin removal Total	DAPT prolongation Events	DAPT prolongation Total	Weight	Risk Ratio M-H, Random, 95% CI
**1.24.1 With oral anticoagulation**						
AUGUSTUS	40	2279	68	2277	43.6%	0.59 [0.40, 0.86]
WOEST	15	279	35	284	19.2%	0.44 [0.24, 0.78]
**Subtotal (95% CI)**		**2558**		**2561**	**62.8%**	**0.54 [0.39, 0.74]**
Total events	55		103			
Heterogeneity: Tau² = 0.00; Chi² = 0.70, df = 1 (P = 0.40); I² = 0%						
Test for overall effect: Z = 3.79 (P = 0.0001)						
**1.24.2 Without oral anticoagulation**						
STOP DAPT 2	6	1500	23	1509	8.1%	0.26 [0.11, 0.64]
TWILIGHT	26	3555	49	3564	29.0%	0.53 [0.33, 0.85]
**Subtotal (95% CI)**		**5055**		**5073**	**37.2%**	**0.42 [0.21, 0.81]**
Total events	32		72			
Heterogeneity: Tau² = 0.12; Chi² = 1.88, df = 1 (P = 0.17); I² = 47%						
Test for overall effect: Z = 2.60 (P = 0.009)						
**Total (95% CI)**		**7613**		**7634**	**100.0%**	**0.51 [0.39, 0.65]**
Total events	87		175			
Heterogeneity: Tau² = 0.00; Chi² = 2.94, df = 3 (P = 0.40); I² = 0%						
Test for overall effect: Z = 5.25 (P < 0.00001)						
Test for subgroup differences: Chi² = 0.46, df = 1 (P = 0.50), I² = 0%						

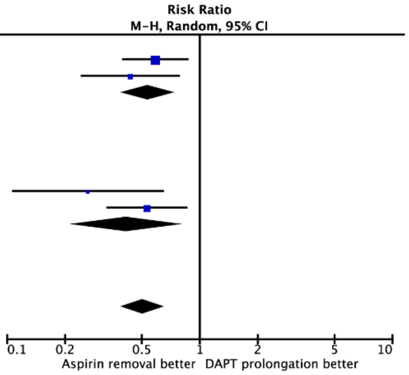

Supplementary Figure S9. Estimated risk of safety events according to the ISTH classification

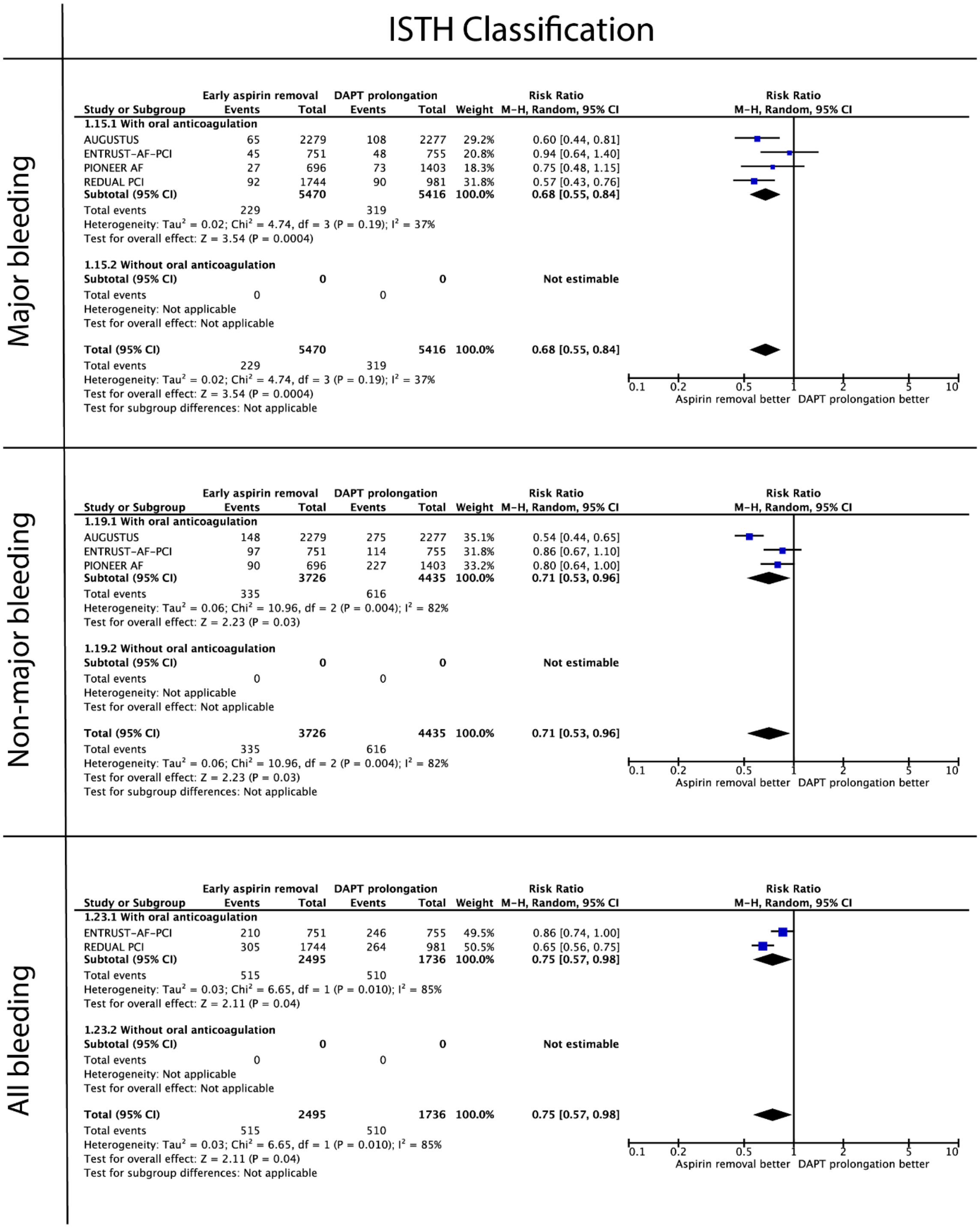

Supplementary Figure S10. Estimated risk of safety event according to the TIMI classification

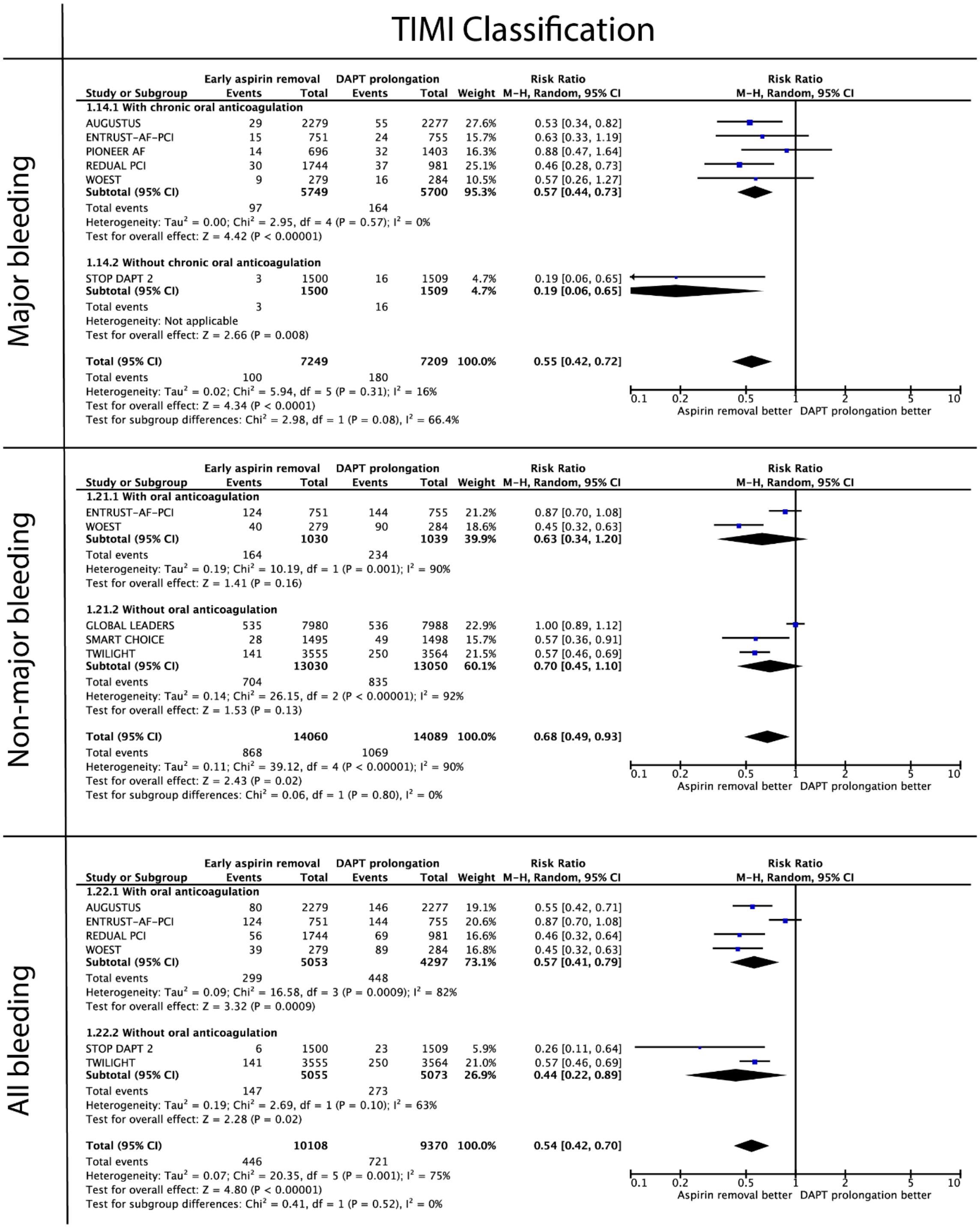

Supplementary Figure S11. Estimated risk of safety event(A) and efficacy events (B) according to the P2Y12 inhibitors predominantly used in trials without indication for chronic oral anticoagulation

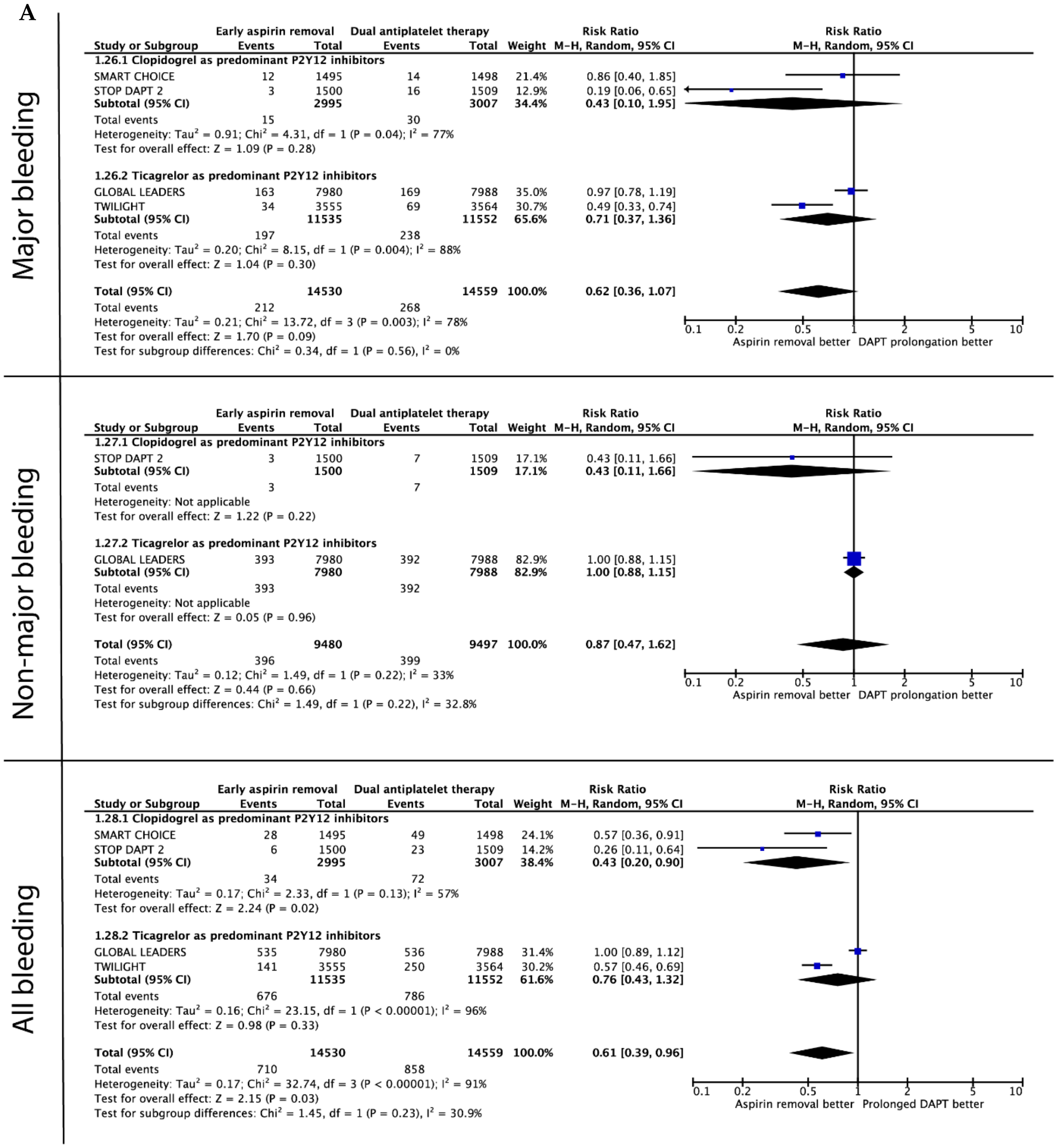

B

All cause death

Study or Subgroup	Early aspirin removal Events	Early aspirin removal Total	Dual antiplatelet therapy Events	Dual antiplatelet therapy Total	Weight	Risk Ratio M-H, Random, 95% CI
**1.28.1 Clopidogrel as predominant P2Y12 inhibitors**						
SMART CHOICE	21	1495	18	1498	6.1%	1.17 [0.63, 2.19]
STOP DAPT 2	21	1500	18	1509	6.1%	1.17 [0.63, 2.19]
**Subtotal (95% CI)**		**2995**		**3007**	**12.1%**	**1.17 [0.75, 1.82]**
Total events	42		36			
Heterogeneity: Tau² = 0.00; Chi² = 0.00, df = 1 (P = 0.99); I² = 0%						
Test for overall effect: Z = 0.70 (P = 0.48)						
**1.28.2 Ticagrelor as predominant P2Y12 inhibitors**						
GLOBAL LEADERS	224	7980	253	7988	75.7%	0.89 [0.74, 1.06]
TWILIGHT	34	3524	45	3515	12.1%	0.75 [0.48, 1.17]
**Subtotal (95% CI)**		**11504**		**11503**	**87.9%**	**0.87 [0.74, 1.02]**
Total events	258		298			
Heterogeneity: Tau² = 0.00; Chi² = 0.44, df = 1 (P = 0.51); I² = 0%						
Test for overall effect: Z = 1.71 (P = 0.09)						
**Total (95% CI)**		**14499**		**14510**	**100.0%**	**0.90 [0.77, 1.05]**
Total events	300		334			
Heterogeneity: Tau² = 0.00; Chi² = 2.01, df = 3 (P = 0.57); I² = 0%						
Test for overall effect: Z = 1.35 (P = 0.18)						
Test for subgroup differences: Chi² = 1.57, df = 1 (P = 0.21), I² = 36.1%						

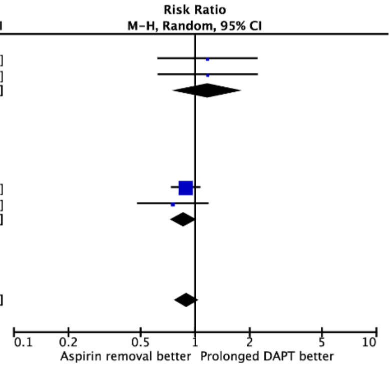

MACCE

Study or Subgroup	Early aspirin removal Events	Early aspirin removal Total	Dual antiplatelet therapy Events	Dual antiplatelet therapy Total	Weight	Risk Ratio M-H, Random, 95% CI
**1.26.1 Clopidogrel as predominant P2Y12 inhibitors**						
SMART CHOICE	42	1495	36	1498	6.4%	1.17 [0.75, 1.81]
STOP DAPT 2	29	1500	37	1509	5.3%	0.79 [0.49, 1.28]
**Subtotal (95% CI)**		**2995**		**3007**	**11.8%**	**0.97 [0.66, 1.43]**
Total events	71		73			
Heterogeneity: Tau² = 0.02; Chi² = 1.40, df = 1 (P = 0.24); I² = 29%						
Test for overall effect: Z = 0.14 (P = 0.89)						
**1.26.2 Ticagrelor as predominant P2Y12 inhibitors**						
GLOBAL LEADERS	362	7980	416	7988	65.5%	0.87 [0.76, 1.00]
TWILIGHT	135	3524	137	3515	22.8%	0.98 [0.78, 1.24]
**Subtotal (95% CI)**		**11504**		**11503**	**88.2%**	**0.90 [0.80, 1.01]**
Total events	497		553			
Heterogeneity: Tau² = 0.00; Chi² = 0.77, df = 1 (P = 0.38); I² = 0%						
Test for overall effect: Z = 1.77 (P = 0.08)						
**Total (95% CI)**		**14499**		**14510**	**100.0%**	**0.91 [0.81, 1.01]**
Total events	568		626			
Heterogeneity: Tau² = 0.00; Chi² = 2.40, df = 3 (P = 0.49); I² = 0%						
Test for overall effect: Z = 1.71 (P = 0.09)						
Test for subgroup differences: Chi² = 0.15, df = 1 (P = 0.70), I² = 0%						

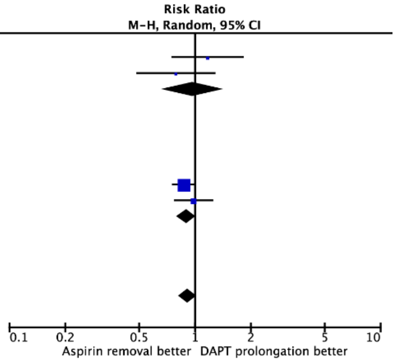

Myocardial infarction

Study or Subgroup	Early aspirin removal Events	Early aspirin removal Total	Dual antiplatelet therapy Events	Dual antiplatelet therapy Total	Weight	Risk Ratio M-H, Random, 95% CI
**1.27.1 Clopidogrel as predominant P2Y12 inhibitors**						
SMART CHOICE	11	1495	17	1498	6.2%	0.65 [0.30, 1.38]
STOP DAPT 2	13	1500	11	1509	5.6%	1.19 [0.53, 2.65]
**Subtotal (95% CI)**		**2995**		**3007**	**11.8%**	**0.86 [0.48, 1.57]**
Total events	24		28			
Heterogeneity: Tau² = 0.03; Chi² = 1.17, df = 1 (P = 0.28); I² = 14%						
Test for overall effect: Z = 0.48 (P = 0.63)						
**1.27.2 Ticagrelor as predominant P2Y12 inhibitors**						
GLOBAL LEADERS	83	7980	103	7988	43.0%	0.81 [0.61, 1.08]
TWILIGHT	95	3524	95	3515	45.2%	1.00 [0.75, 1.32]
**Subtotal (95% CI)**		**11504**		**11503**	**88.2%**	**0.90 [0.73, 1.11]**
Total events	178		198			
Heterogeneity: Tau² = 0.00; Chi² = 1.07, df = 1 (P = 0.30); I² = 7%						
Test for overall effect: Z = 1.00 (P = 0.32)						
**Total (95% CI)**		**14499**		**14510**	**100.0%**	**0.89 [0.74, 1.08]**
Total events	202		226			
Heterogeneity: Tau² = 0.00; Chi² = 2.26, df = 3 (P = 0.52); I² = 0%						
Test for overall effect: Z = 1.15 (P = 0.25)						
Test for subgroup differences: Chi² = 0.01, df = 1 (P = 0.90), I² = 0%						

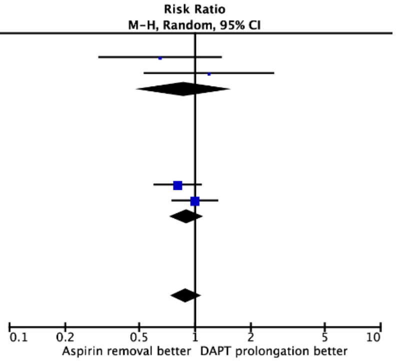

Any stroke

Study or Subgroup	Early aspirin removal Events	Early aspirin removal Total	Dual antiplatelet therapy Events	Dual antiplatelet therapy Total	Weight	Risk Ratio M-H, Random, 95% CI
**1.29.1 Clopidogrel as predominant P2Y12 inhibitors**						
SMART CHOICE	11	1495	5	1498	21.6%	2.20 [0.77, 6.33]
STOP DAPT 2	8	1500	16	1509	27.8%	0.50 [0.22, 1.17]
**Subtotal (95% CI)**		**2995**		**3007**	**49.4%**	**1.02 [0.24, 4.32]**
Total events	19		21			
Heterogeneity: Tau² = 0.85; Chi² = 4.59, df = 1 (P = 0.03); I² = 78%						
Test for overall effect: Z = 0.02 (P = 0.98)						
**1.29.2 Ticagrelor as predominant P2Y12 inhibitors**						
GLOBAL LEADERS	80	7980	82	7988	50.6%	0.98 [0.72, 1.33]
TWILIGHT	0	0	0	0		Not estimable
**Subtotal (95% CI)**		**7980**		**7988**	**50.6%**	**0.98 [0.72, 1.33]**
Total events	80		82			
Heterogeneity: Not applicable						
Test for overall effect: Z = 0.15 (P = 0.88)						
**Total (95% CI)**		**10975**		**10995**	**100.0%**	**0.97 [0.52, 1.80]**
Total events	99		103			
Heterogeneity: Tau² = 0.17; Chi² = 4.64, df = 2 (P = 0.10); I² = 57%						
Test for overall effect: Z = 0.10 (P = 0.92)						
Test for subgroup differences: Chi² = 0.00, df = 1 (P = 0.96), I² = 0%						

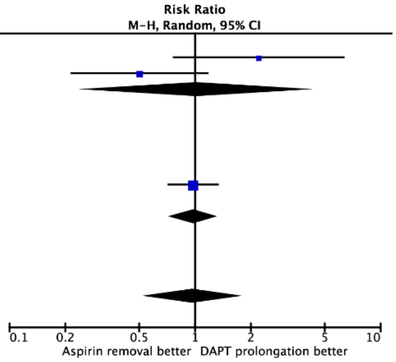

Supplementary Figure S12. Estimated risk of safety event(A) and efficacy events (B) according to the DAPT duration prior to aspirin discontinuation in trials without indication for chronic oral anticoagulation

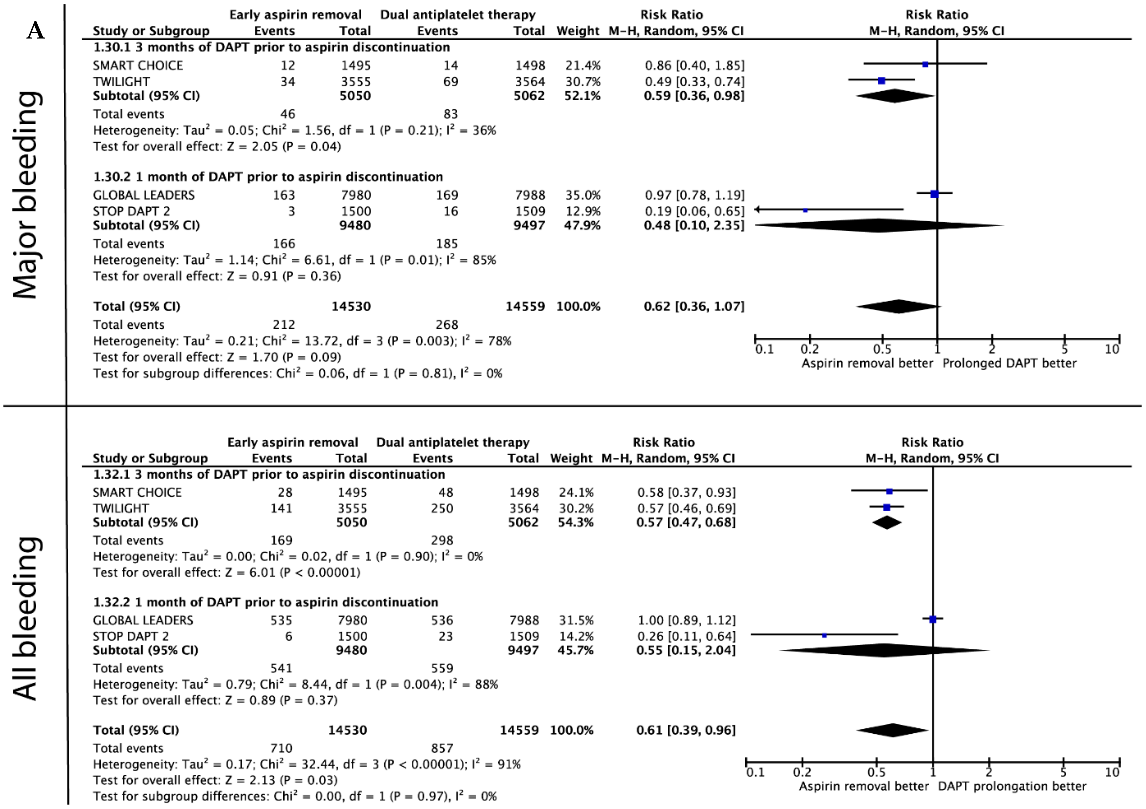

B

All cause death

Study or Subgroup	Early aspirin removal Events	Early aspirin removal Total	Dual antiplatelet therapy Events	Dual antiplatelet therapy Total	Weight	Risk Ratio M–H, Random, 95% CI
**1.28.1 Clopidogrel as predominant P2Y12 inhibitors**						
SMART CHOICE	21	1495	18	1498	6.1%	1.17 [0.63, 2.19]
STOP DAPT 2	21	1500	18	1509	6.1%	1.17 [0.63, 2.19]
**Subtotal (95% CI)**		**2995**		**3007**	**12.1%**	**1.17 [0.75, 1.82]**
Total events	42		36			
Heterogeneity: Tau^2 = 0.00; Chi^2 = 0.00, df = 1 (P = 0.99); I^2 = 0%						
Test for overall effect: Z = 0.70 (P = 0.48)						
**1.28.2 Ticagrelor as predominant P2Y12 inhibitors**						
GLOBAL LEADERS	224	7980	253	7988	75.7%	0.89 [0.74, 1.06]
TWILIGHT	34	3524	45	3515	12.1%	0.75 [0.48, 1.17]
**Subtotal (95% CI)**		**11504**		**11503**	**87.9%**	**0.87 [0.74, 1.02]**
Total events	258		298			
Heterogeneity: Tau^2 = 0.00; Chi^2 = 0.44, df = 1 (P = 0.51); I^2 = 0%						
Test for overall effect: Z = 1.71 (P = 0.09)						
**Total (95% CI)**		**14499**		**14510**	**100.0%**	**0.90 [0.77, 1.05]**
Total events	300		334			
Heterogeneity: Tau^2 = 0.00; Chi^2 = 2.01, df = 3 (P = 0.57); I^2 = 0%						
Test for overall effect: Z = 1.35 (P = 0.18)						
Test for subgroup differences: Chi^2 = 1.57, df = 1 (P = 0.21), I^2 = 36.1%						

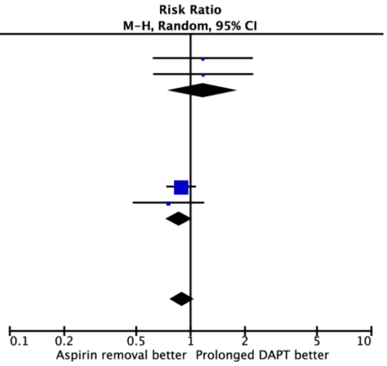

MACCE

Study or Subgroup	Early aspirin removal Events	Early aspirin removal Total	Dual antiplatelet therapy Events	Dual antiplatelet therapy Total	Weight	Risk Ratio M–H, Random, 95% CI
**1.35.1 3 months of DAPT prior to aspirin discontinuation**						
SMART CHOICE	42	1495	36	1498	6.4%	1.17 [0.75, 1.81]
TWILIGHT	135	3524	137	3515	22.8%	0.98 [0.78, 1.24]
**Subtotal (95% CI)**		**5019**		**5013**	**29.2%**	**1.02 [0.83, 1.25]**
Total events	177		173			
Heterogeneity: Tau^2 = 0.00; Chi^2 = 0.47, df = 1 (P = 0.49); I^2 = 0%						
Test for overall effect: Z = 0.20 (P = 0.84)						
**1.35.2 1 month of DAPT prior to aspirin discontinuation**						
GLOBAL LEADERS	362	7980	416	7988	65.5%	0.87 [0.76, 1.00]
STOP DAPT 2	29	1500	37	1509	5.3%	0.79 [0.49, 1.28]
**Subtotal (95% CI)**		**9480**		**9497**	**70.8%**	**0.86 [0.76, 0.99]**
Total events	391		453			
Heterogeneity: Tau^2 = 0.00; Chi^2 = 0.15, df = 1 (P = 0.70); I^2 = 0%						
Test for overall effect: Z = 2.16 (P = 0.03)						
**Total (95% CI)**		**14499**		**14510**	**100.0%**	**0.91 [0.81, 1.01]**
Total events	568		626			
Heterogeneity: Tau^2 = 0.00; Chi^2 = 2.40, df = 3 (P = 0.49); I^2 = 0%						
Test for overall effect: Z = 1.71 (P = 0.09)						
Test for subgroup differences: Chi^2 = 1.78, df = 1 (P = 0.18), I^2 = 43.7%						

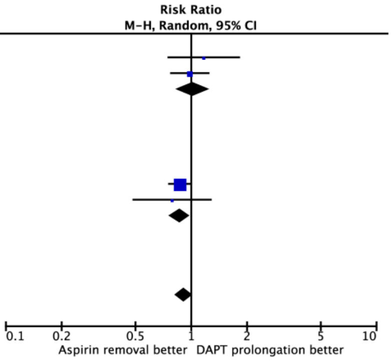

Myocardial infarction

Study or Subgroup	Early aspirin removal Events	Early aspirin removal Total	Dual antiplatelet therapy Events	Dual antiplatelet therapy Total	Weight	Risk Ratio M–H, Random, 95% CI
**1.34.1 3 months of DAPT prior to aspirin discontinuation**						
SMART CHOICE	11	1495	17	1498	6.2%	0.65 [0.30, 1.38]
TWILIGHT	95	3524	95	3515	45.2%	1.00 [0.75, 1.32]
**Subtotal (95% CI)**		**5019**		**5013**	**51.4%**	**0.93 [0.69, 1.27]**
Total events	106		112			
Heterogeneity: Tau^2 = 0.01; Chi^2 = 1.10, df = 1 (P = 0.29); I^2 = 9%						
Test for overall effect: Z = 0.45 (P = 0.66)						
**1.34.2 1 month of DAPT prior to aspirin discontinuation**						
GLOBAL LEADERS	83	7980	103	7988	43.0%	0.81 [0.61, 1.08]
STOP DAPT 2	13	1500	11	1509	5.6%	1.19 [0.53, 2.65]
**Subtotal (95% CI)**		**9480**		**9497**	**48.6%**	**0.84 [0.64, 1.11]**
Total events	96		114			
Heterogeneity: Tau^2 = 0.00; Chi^2 = 0.80, df = 1 (P = 0.37); I^2 = 0%						
Test for overall effect: Z = 1.24 (P = 0.22)						
**Total (95% CI)**		**14499**		**14510**	**100.0%**	**0.89 [0.74, 1.08]**
Total events	202		226			
Heterogeneity: Tau^2 = 0.00; Chi^2 = 2.26, df = 3 (P = 0.52); I^2 = 0%						
Test for overall effect: Z = 1.15 (P = 0.25)						
Test for subgroup differences: Chi^2 = 0.24, df = 1 (P = 0.63), I^2 = 0%						

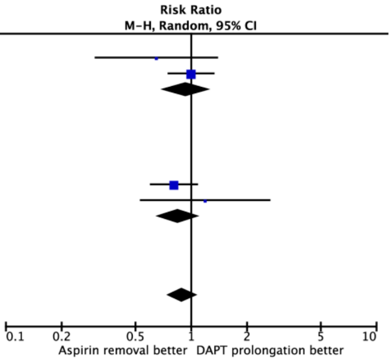

Any stroke

Study or Subgroup	Early aspirin removal Events	Early aspirin removal Total	Dual antiplatelet therapy Events	Dual antiplatelet therapy Total	Weight	Risk Ratio M–H, Random, 95% CI
**1.36.1 3 months of DAPT prior to aspirin discontinuation**						
SMART CHOICE	11	1495	5	1498	21.6%	2.20 [0.77, 6.33]
TWILIGHT	0	0	0	0		Not estimable
**Subtotal (95% CI)**		**1495**		**1498**	**21.6%**	**2.20 [0.77, 6.33]**
Total events	11		5			
Heterogeneity: Not applicable						
Test for overall effect: Z = 1.47 (P = 0.14)						
**1.36.2 1 month of DAPT prior to aspirin discontinuation**						
GLOBAL LEADERS	80	7980	82	7988	50.6%	0.98 [0.72, 1.33]
STOP DAPT 2	8	1500	16	1509	27.8%	0.50 [0.22, 1.17]
**Subtotal (95% CI)**		**9480**		**9497**	**78.4%**	**0.79 [0.43, 1.45]**
Total events	88		98			
Heterogeneity: Tau^2 = 0.11; Chi^2 = 2.09, df = 1 (P = 0.15); I^2 = 52%						
Test for overall effect: Z = 0.76 (P = 0.45)						
**Total (95% CI)**		**10975**		**10995**	**100.0%**	**0.97 [0.52, 1.80]**
Total events	99		103			
Heterogeneity: Tau^2 = 0.17; Chi^2 = 4.64, df = 2 (P = 0.10); I^2 = 57%						
Test for overall effect: Z = 0.10 (P = 0.92)						
Test for subgroup differences: Chi^2 = 2.73, df = 1 (P = 0.10), I^2 = 63.3%						

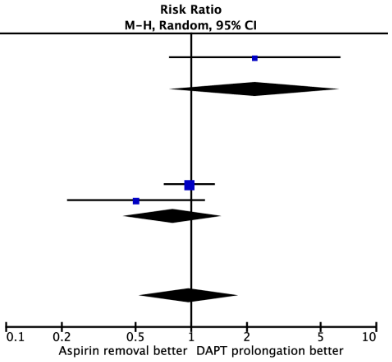

Supplementary Figure S13. Estimated risk of efficacy (A) and safety (B) events using adjudicated data from GLASSY trial

A

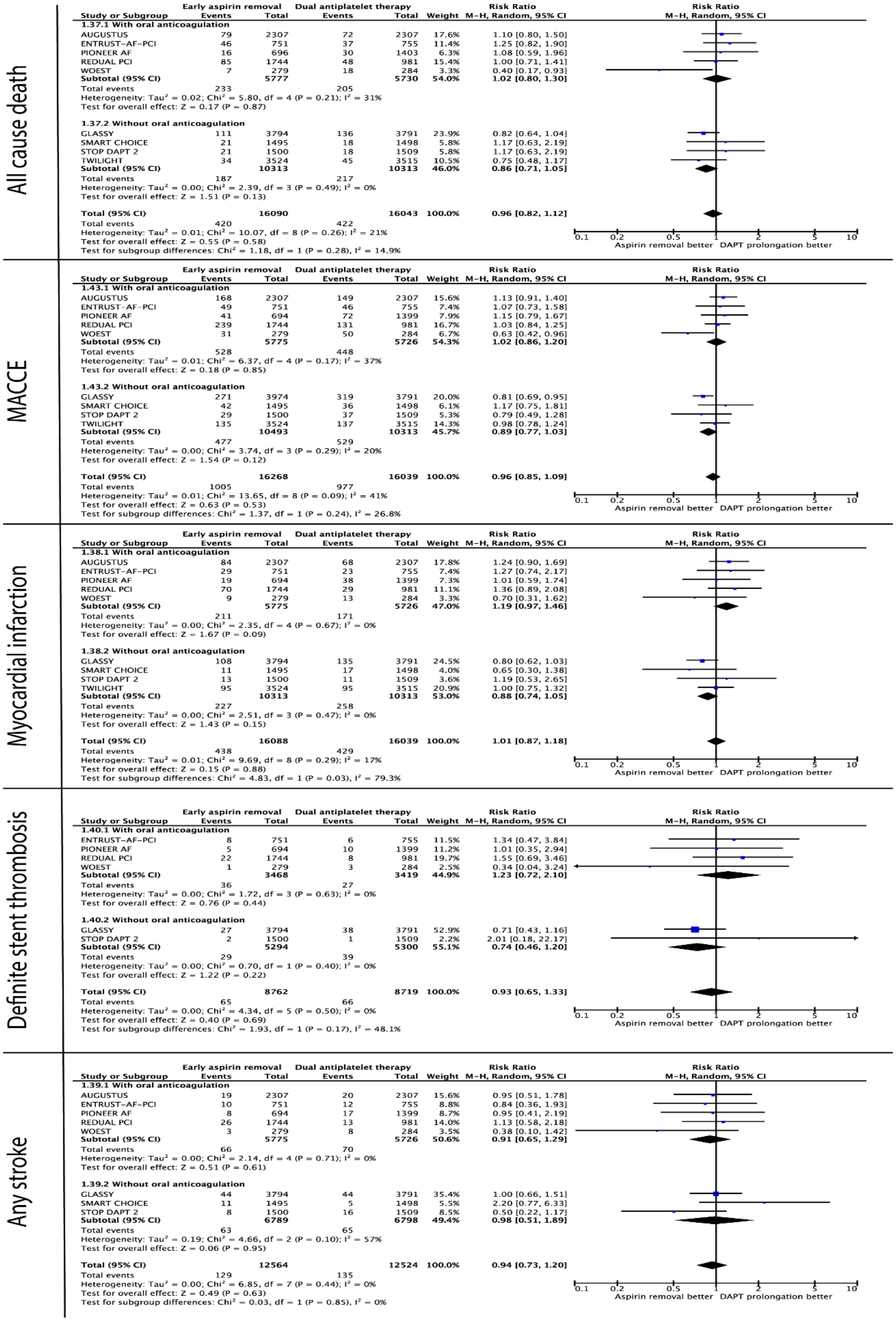

B

Major bleeding

Study or Subgroup	Early aspirin removal Events	Early aspirin removal Total	Dual antiplatelet therapy Events	Dual antiplatelet therapy Total	Weight	Risk Ratio M-H, Random, 95% CI
**1.41.1 With oral anticoagulation**						
AUGUSTUS	65	2279	108	2277	16.0%	0.60 [0.44, 0.81]
ENTRUST-AF-PCI	45	751	48	755	13.5%	0.94 [0.64, 1.40]
PIONEER AF	14	696	32	1403	8.6%	0.88 [0.47, 1.64]
REDUAL PCI	92	1744	90	981	16.6%	0.57 [0.43, 0.76]
WOEST	9	279	16	284	6.1%	0.57 [0.26, 1.27]
**Subtotal (95% CI)**		**5749**		**5700**	**60.8%**	**0.67 [0.55, 0.83]**
Total events	225		294			
Heterogeneity: $Tau^2 = 0.01$; $Chi^2 = 5.40$, df = 4 (P = 0.25); $I^2 = 26\%$						
Test for overall effect: Z = 3.67 (P = 0.0002)						
**1.41.2 Without oral anticoagulation**						
GLASSY	94	3794	94	3791	16.6%	1.00 [0.75, 1.33]
SMART CHOICE	12	1495	14	1498	6.5%	0.86 [0.40, 1.85]
STOP DAPT 2	3	1500	16	1509	3.1%	0.19 [0.06, 0.65]
TWILIGHT	34	3555	69	3564	13.1%	0.49 [0.33, 0.74]
**Subtotal (95% CI)**		**10344**		**10362**	**39.2%**	**0.62 [0.35, 1.09]**
Total events	143		193			
Heterogeneity: $Tau^2 = 0.23$; $Chi^2 = 12.98$, df = 3 (P = 0.005); $I^2 = 77\%$						
Test for overall effect: Z = 1.65 (P = 0.10)						
**Total (95% CI)**		**16093**		**16062**	**100.0%**	**0.68 [0.54, 0.86]**
Total events	368		487			
Heterogeneity: $Tau^2 = 0.06$; $Chi^2 = 19.38$, df = 8 (P = 0.01); $I^2 = 59\%$						
Test for overall effect: Z = 3.19 (P = 0.001)						
Test for subgroup differences: $Chi^2 = 0.07$, df = 1 (P = 0.79), $I^2 = 0\%$						

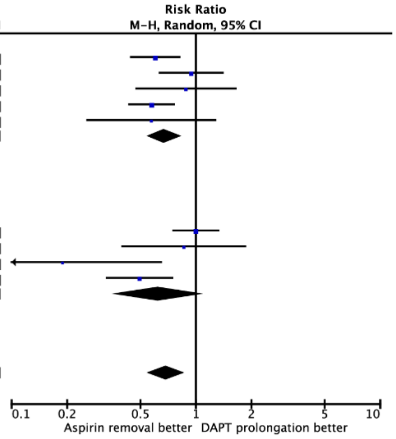

Non-major bleeding

Study or Subgroup	Early aspirin removal Events	Early aspirin removal Total	Dual antiplatelet therapy Events	Dual antiplatelet therapy Total	Weight	Risk Ratio M-H, Random, 95% CI
**1.42.1 With oral anticoagulation**						
AUGUSTUS	148	2279	275	2277	22.8%	0.54 [0.44, 0.65]
ENTRUST-AF-PCI	97	751	114	755	21.7%	0.86 [0.67, 1.10]
PIONEER AF	7	696	20	1403	9.8%	0.71 [0.30, 1.66]
WOEST	23	279	59	284	17.3%	0.40 [0.25, 0.62]
**Subtotal (95% CI)**		**4005**		**4719**	**71.7%**	**0.60 [0.42, 0.85]**
Total events	275		468			
Heterogeneity: $Tau^2 = 0.08$; $Chi^2 = 12.24$, df = 3 (P = 0.007); $I^2 = 75\%$						
Test for overall effect: Z = 2.88 (P = 0.004)						
**1.42.2 Without oral anticoagulation**						
GLASSY	244	3794	239	3791	23.1%	1.02 [0.86, 1.21]
STOP DAPT 2	3	1500	7	1509	5.2%	0.43 [0.11, 1.66]
**Subtotal (95% CI)**		**5294**		**5300**	**28.3%**	**0.87 [0.45, 1.68]**
Total events	247		246			
Heterogeneity: $Tau^2 = 0.13$; $Chi^2 = 1.54$, df = 1 (P = 0.21); $I^2 = 35\%$						
Test for overall effect: Z = 0.42 (P = 0.68)						
**Total (95% CI)**		**9299**		**10019**	**100.0%**	**0.66 [0.47, 0.94]**
Total events	522		714			
Heterogeneity: $Tau^2 = 0.13$; $Chi^2 = 33.17$, df = 5 (P < 0.00001); $I^2 = 85\%$						
Test for overall effect: Z = 2.32 (P = 0.02)						
Test for subgroup differences: $Chi^2 = 0.97$, df = 1 (P = 0.32), $I^2 = 0\%$						

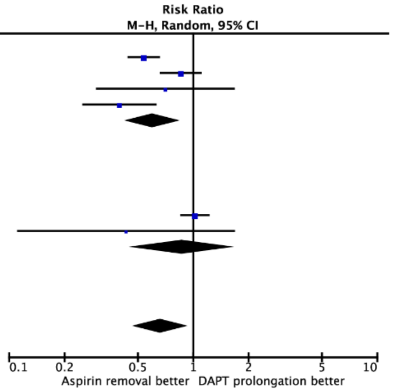

Supplementary Figure S14. Estimated risk of Myocardial infarction using site-reported events with the GLOBAL LEADERS trial.

	Experimental		Control			Risk Ratio
**Study or Subgroup**	**Events**	**Total**	**Events**	**Total**	**Weight**	**M-H, Random, 95% CI**
**1.28.1 With oral anticoagulation**						
AUGUSTUS	84	2307	68	2307	13.8%	1.24 [0.90, 1.69]
ENTRUST-AF-PCI	29	751	23	755	4.7%	1.27 [0.74, 2.17]
PIONEER AF	19	694	38	1399	4.6%	1.01 [0.59, 1.74]
REDUAL PCI	70	1744	29	981	7.5%	1.36 [0.89, 2.08]
WOEST	9	279	13	284	2.0%	0.70 [0.31, 1.62]
**Subtotal (95% CI)**		**5775**		**5726**	**32.6%**	**1.19 [0.97, 1.46]**
Total events	211		171			
Heterogeneity: Tau² = 0.00; Chi² = 2.35, df = 4 (P = 0.67); I² = 0%						
Test for overall effect: Z = 1.67 (P = 0.09)						
**1.28.2 Without oral anticoagulation**						
GLOBAL LEADERS	248	7980	250	7988	45.6%	0.99 [0.84, 1.18]
SMART CHOICE	11	1495	17	1498	2.4%	0.65 [0.30, 1.38]
STOP DAPT 2	13	1500	11	1509	2.1%	1.19 [0.53, 2.65]
TWILIGHT	95	3524	95	3515	17.3%	1.00 [0.75, 1.32]
**Subtotal (95% CI)**		**14499**		**14510**	**67.4%**	**0.98 [0.85, 1.14]**
Total events	367		373			
Heterogeneity: Tau² = 0.00; Chi² = 1.41, df = 3 (P = 0.70); I² = 0%						
Test for overall effect: Z = 0.21 (P = 0.83)						
**Total (95% CI)**		**20274**		**20236**	**100.0%**	**1.05 [0.93, 1.18]**
Total events	578		544			
Heterogeneity: Tau² = 0.00; Chi² = 5.98, df = 8 (P = 0.65); I² = 0%						
Test for overall effect: Z = 0.78 (P = 0.44)						
Test for subgroup differences: Chi² = 2.23, df = 1 (P = 0.14), I² = 55.1%						

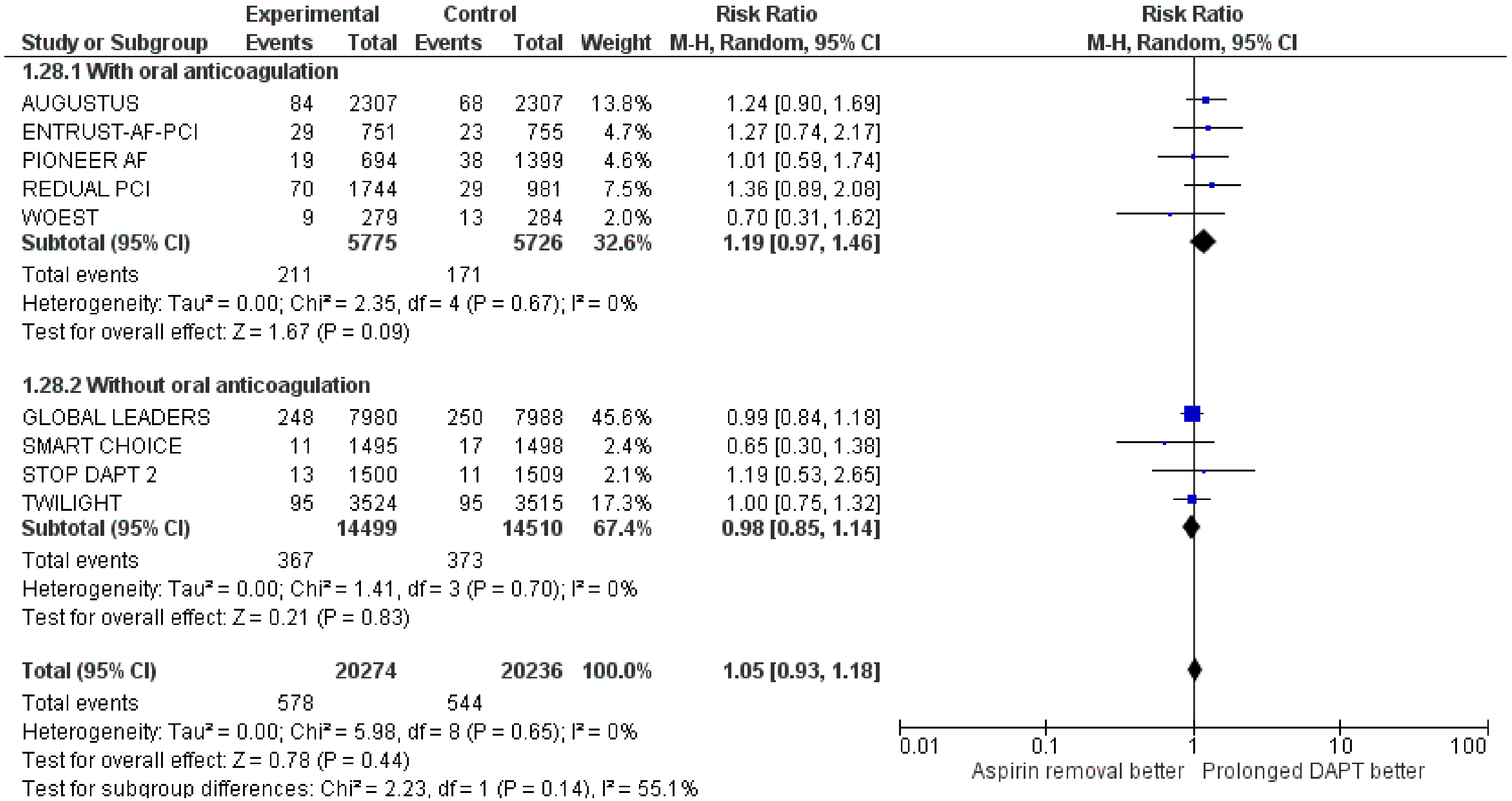

Supplementary Figure S15. Funnel plots for each outcome

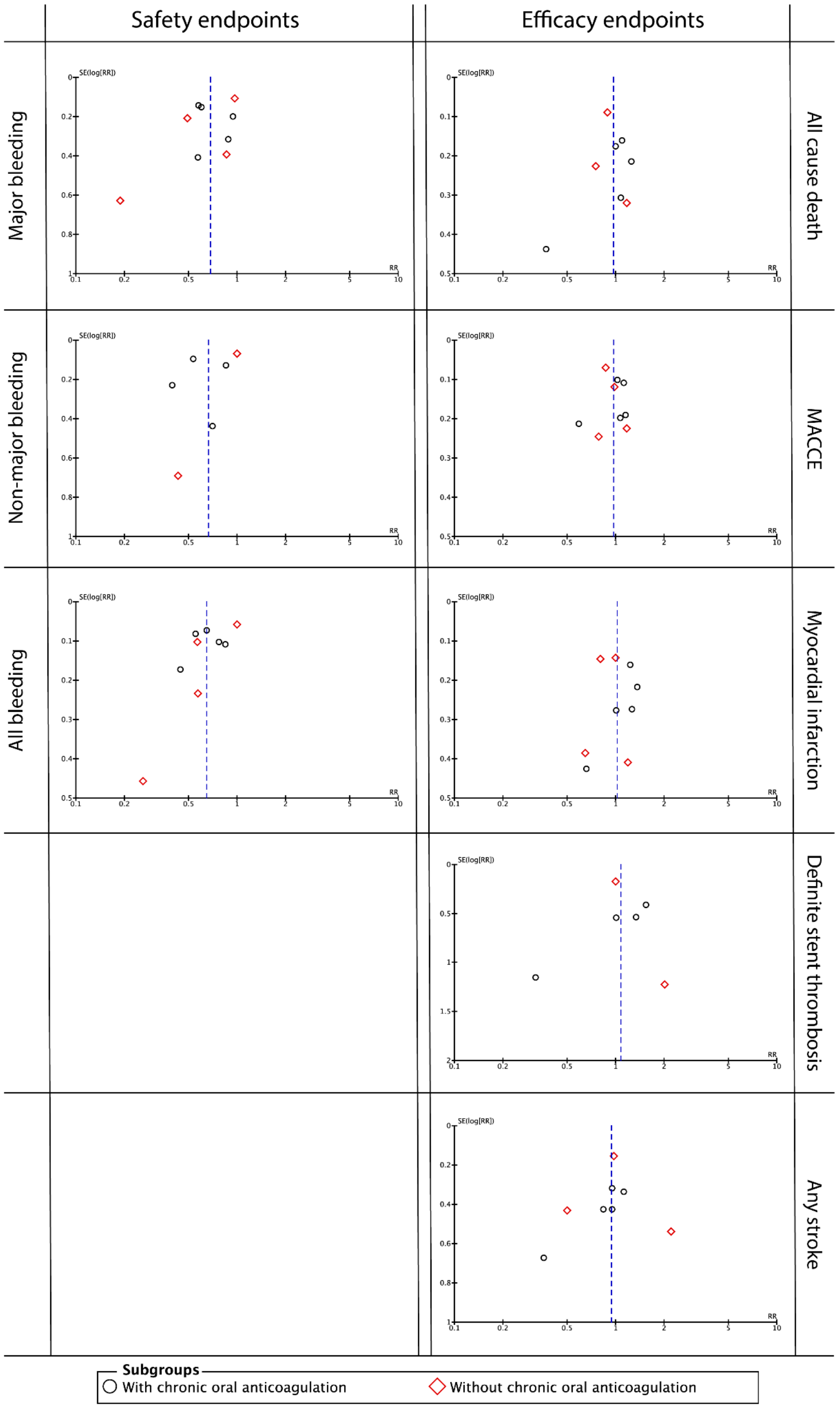

Online References

1. Vranckx, P.; Valgimigli, M.; Jüni, P.; Hamm, C.; Steg, P.G.; Heg, D.; van Es, G.A.; McFadden, E.P.; Onuma, Y.; van Meijeren, C.; et al. Ticagrelor plus aspirin for 1 month, followed by ticagrelor monotherapy for 23 months vs aspirin plus clopidogrel or ticagrelor for 12 months, followed by aspirin monotherapy for 12 months after implantation of a drug-eluting stent: a multicentre, open-label, randomised superiority trial. *Lancet* **2018**, *392*, 940–949.
2. Watanabe, H.; Domei, T.; Morimoto, T.; Natsuaki, M.; Shiomi, H.; Toyota, T.; Ohya, M.; Suwa, S.; Takagi, K.; Nanasato, M.; et al. Effect of 1-Month Dual Antiplatelet Therapy Followed by Clopidogrel vs 12-Month Dual Antiplatelet Therapy on Cardiovascular and Bleeding Events in Patients Receiving PCI: The STOPDAPT-2 Randomized Clinical Trial. *JAMA* **2019**, *321*, 2414–2427.
3. Hahn, J.-Y.; Song, Y.B.; Oh, J.-H.; Chun, W.J.; Park, Y.H.; Jang, W.J.; Im, E.-S.; Jeong, J.-O.; Cho, B.R.; Oh, S.K.; et al. Effect of P2Y12 Inhibitor Monotherapy vs Dual Antiplatelet Therapy on Cardiovascular Events in Patients Undergoing Percutaneous Coronary Intervention: The SMART-CHOICE Randomized Clinical Trial. *JAMA* **2019**, *321*, 2428–2437.
4. Dewilde, W.J.M.; Oirbans, T.; Verheugt, F.W.A.; Kelder, J.C.; De Smet, B.J.G.L.; Herrman, J.-P.; Adriaenssens, T.; Vrolix, M.; Heestermans, A.A.C.M.; Vis, M.M.; et al. Use of clopidogrel with or without aspirin in patients taking oral anticoagulant therapy and undergoing percutaneous coronary intervention: an open-label, randomised, controlled trial. *Lancet* **2013**, *381*, 1107–1115.
5. Lopes, R.D.; Heizer, G.; Aronson, R.; Vora, A.N.; Massaro, T.; Mehran, R.; Goodman, S.G.; Windecker, S.; Darius, H.; Li, J.; et al. Antithrombotic Therapy after Acute Coronary Syndrome or PCI in Atrial Fibrillation. *N. Engl. J. Med.* **2019**, *380*, 1509–1524.
6. Mehran, R.; Baber, U.; Sharma, S.K.; Cohen, D.J.; Angiolillo, D.J.; Briguori, C.; Cha, J.Y.; Collier, T.; Dangas, G.; Dudek, D.; et al. Ticagrelor with or without Aspirin in High-Risk Patients after PCI. *N. Engl. J. Med.* **2019**.
7. Cannon, C.P.; Bhatt, D.L.; Oldgren, J.; Lip, G.Y.H.; Ellis, S.G.; Kimura, T.; Maeng, M.; Merkely, B.; Zeymer, U.; Gropper, S.; et al. Dual Antithrombotic Therapy with Dabigatran after PCI in Atrial Fibrillation. *N. Engl. J. Med.* **2017**, *377*, 1513–1524.
8. Gibson, C.M.; Mehran, R.; Bode, C.; Halperin, J.; Verheugt, F.W.; Wildgoose, P.; Birmingham, M.; Ianus, J.; Burton, P.; van Eickels, M.; et al. Prevention of Bleeding in Patients with Atrial Fibrillation Undergoing PCI. *N. Engl. J. Med.* **2016**, *375*, 2423–2434.
9. Vranckx, P.; Valgimigli, M.; Eckardt, L.; Tijssen, J.; Lewalter, T.; Gargiulo, G.; Batushkin, V.; Campo, G.; Lysak, Z.; Vakaliuk, I.; et al. Edoxaban-based versus vitamin K antagonist-based antithrombotic regimen after successful coronary stenting in patients with atrial fibrillation (ENTRUST-AF PCI): a randomised, open-label, phase 3b trial. *Lancet* **2019**.
